# Evolution of Alternative Splicing in Eudicots

**DOI:** 10.3389/fpls.2019.00707

**Published:** 2019-06-12

**Authors:** Zhihao Ling, Thomas Brockmöller, Ian T. Baldwin, Shuqing Xu

**Affiliations:** ^1^Max Planck Institute for Chemical Ecology, Jena, Germany; ^2^Plant Adaptation-in-action Group, Institute for Evolution and Biodiversity, University of Münster, Münster, Germany

**Keywords:** alternative splicing, evolution, transcriptome, splicing code, deep learning, nonsense-mediated decay

## Abstract

Alternative pre-mRNA splicing (AS) is prevalent in plants and is involved in many interactions between plants and environmental stresses. However, the patterns and underlying mechanisms of AS evolution in plants remain unclear. By analyzing the transcriptomes of four eudicot species, we revealed that the divergence of AS is largely due to the gains and losses of AS events among orthologous genes. Furthermore, based on a subset of AS, in which AS can be directly associated with specific transcripts, we found that AS that generates transcripts containing premature termination codons (PTC), are likely more conserved than those that generate non-PTC containing transcripts. This suggests that AS coupled with nonsense-mediated decay (NMD) might play an important role in affecting mRNA levels post-transcriptionally. To understand the mechanisms underlying the divergence of AS, we analyzed the key determinants of AS using a machine learning approach. We found that the presence/absence of alternative splice site (SS) within the junction, the distance between the authentic SS and the nearest alternative SS, the size of exon–exon junctions were the major determinants for both alternative 5′ donor site and 3′ acceptor site among the studied species, suggesting a relatively conserved AS mechanism. The comparative analysis further demonstrated that variations of the identified AS determinants significantly contributed to the AS divergence among closely related species in both Solanaceae and Brassicaceae taxa. Together, these results provide detailed insights into the evolution of AS in plants.

## Introduction

Due to their sessile lifestyle, plants have evolved various mechanisms to respond to environmental stresses. Alternative splicing (AS), a mechanism by which different mature RNAs are formed by removing different introns or using different splice sites (SS) from the same pre-mRNA, is known to be important for stress-induced responses in plants (Mastrangelo et al., 2012; Staiger and Brown, 2013). Both biotic and abiotic stresses such as herbivores (Ling et al., 2015), pathogens (Howard et al., 2013), and cold and salt (Ding et al., 2014) can all induce genome-wide changes in AS in plants. The environment-induced AS changes in turn can affect phenotypic traits of plants and may contribute to their adaptations to different stresses (Mastrangelo et al., 2012; Staiger and Brown, 2013). For example, low temperature-induced AS changes of flowering regulator genes affect flowering time and floral development in *Arabidopsis thaliana* (Severing et al., 2012; Rosloski et al., 2013). The strong association between AS and environmental stimuli suggests that AS is involved in adaptation processes and thus may have evolved rapidly.

Two main functions of AS have been postulated: (i) to expand proteome diversity when different transcript isoforms are translated into different proteins (with different subcellular localization, stability, enzyme activity etc.) (Kazan, 2003; Reddy, 2007; Barbazuk et al., 2008); (ii) to influence gene expression (GE) by generating transcripts harboring premature termination codons (PTC) that are recognized by the nonsense-mediated decay (NMD) machinery and degraded (Chang et al., 2007; Hori and Watanabe, 2007; Kalyna et al., 2012; Kervestin and Jacobson, 2012). For example, different environmental stresses can induce AS events that generate PTC containing (+PTC) transcripts in key splicing regulators and circadian genes (Palusa et al., 2007; Filichkin et al., 2010, 2015). Although initially considered to be transcriptional noise, several AS events that introduce PTCs have been found to be highly conserved in animals (Ni et al., 2007; Lareau and Brenner, 2015) and plants (Iida and Go, 2006; Kalyna et al., 2006; Darracq and Adams, 2013), suggesting that that the combination of AS with NMD might play an important role in affecting mRNA levels post-transcriptionally. However, it is unclear whether NMD-coupled AS is more conserved than the AS that generates transcripts without PTC at the genome-wide level.

The evolution of AS in plants, compared to that in vertebrates, remains largely unclear. Studies that compared organ-specific transcriptomes from different vertebrate species spanning ∼350 million years of evolution showed that AS complexity differs dramatically among vertebrate lineages, and AS evolved much faster than GE (Barbosa-Morais et al., 2012; Merkin et al., 2012). For example, within 6 million years, the splicing profiles of an organ are more similar to other organs of the same species than the same organ in other species, while the expression profiles of the same organ are similar to the organ in other species (Barbosa-Morais et al., 2012; Merkin et al., 2012). In plants, largely due to the lack of comprehensive transcriptomic data, such comparative analysis remains unavailable. However, several indications suggest that AS in plants and vertebrates may share a similar evolution pattern. For example, only 16.4% of AS between maize and rice, and 5.4% between *Brassica* and *Arabidopsis* are conserved (Severing et al., 2009; Darracq and Adams, 2013). A more recent study further showed that only 2.8% of genes showed conserved AS between two species of mung beans, *Vigna radiata* and *V. angularis* (Satyawan et al., 2016). Furthermore, large changes in AS also exist between different ecotypes of the same species (Ner-Gaon et al., 2007). However, such low conservation of AS found among species could also be due to several other confounding effects. For example, it is also known that the levels of gene expression, which are highly associated with AS, also diverge rapidly in plants (Yang and Wang, 2013). As a consequence, it remains unclear whether the low observed levels of AS conservation are resulted from the rapid expression changes between species. Additionally, AS detection is highly dependent on sequencing depth and the tissue types used for generating transcriptomic data (Xu et al., 2002; Ellis et al., 2012; Ling et al., 2015). Therefore, it is necessary to systematically control for different confounding effects in order to understand the evolutionary patterns of AS in plants.

From a mechanistic perspective, the divergence of AS among species is affected by factors that affect the exon-intron splicing process, which is mediated by the spliceosome. While the recognition processes of exonic and intronic regions are directed by sequence features of the pre-mRNA in animals, how the spliceosome removes introns and ligates exons is poorly understood in plants. In metazoans, it is known that four crucial signals are required for accurate splicing: (i) 5′ SS, which contains a GU dinucleotide at the intron start surrounded by a piece of longer consensus sequences of lower conservation, (ii) 3′ SS, which includes an AG dinucleotide at the 3′ end surrounded by similar sequences of 5′ SS, (iii) a polypyrimidine tract and (iv) a branch site sequence located ∼17–40 nt upstream of the 3′ SS (Black, 2003; Fu and Ares, 2014). In plants, similar sequence features with a small difference at specific positions were found, except for the requirement of a branch site (Reddy, 2007). In addition, a UA-rich tract in introns has also been found to be important for efficient splicing in plants (Lewandowska et al., 2004; Simpson et al., 2004; Baek et al., 2008). In animals, the regulation of splicing also depends largely on *cis* signals and *trans*-acting splicing factors that can recognize the signals (Barbosa-Morais et al., 2012; Merkin et al., 2012). Among different splicing factors, serine/arginine-rich (SR) proteins are from an important splicing factor family that has been shown to be involved in AS regulation (Lopato et al., 1999; Gao et al., 2004; Wang and Brendel, 2004; Reddy, 2007; Reddy and Shad Ali, 2011). In addition, many splicing regulatory elements (SREs) and RNA-binding proteins (RBPs) have been identified in animals, and the interactions among these SREs in the pre-mRNA and RBPs were found either to promote or suppress the use of particular SS (Licatalosi et al., 2008; Chen and Manley, 2009; Barash et al., 2010). The number of SR proteins genes in plants (on average > 20) is nearly twice of the number found in non-photosynthetic organisms, although the number varies among different species (Iida and Go, 2006; Isshiki et al., 2006; Richardson et al., 2011). To date, more than 1,000 RBPs and 80 SREs have been identified in plants using computational approaches (Lorkovic, 2009; Marondedze et al., 2016), however, only a few of these have been functionally validated (Yoshimura et al., 2002; Pertea et al., 2007; Schoning et al., 2008; Thomas et al., 2012).

In mammals, the emergence of AS originated from constitutive splicing with the fixation of SREs and the creation of alternative competing SS (Koren et al., 2007; Lev-Maor et al., 2007). Distinctive features that distinguish alternatively spliced exons/introns from constitutively spliced exons/introns can be used to accurately predict the specific AS type (Koren et al., 2007; Braunschweig et al., 2014). Furthermore, other factors including secondary and tertiary RNA structures, chromatin remodeling, insertion of transposable elements (TEs) and gene duplication may also be involved in regulating AS (Liu et al., 1995; Sorek et al., 2002; Donahue et al., 2006; Su et al., 2006; Kolasinska-Zwierz et al., 2009; Schwartz et al., 2009; Warf and Berglund, 2010; Lambert et al., 2015). However, the extent to which changes in these factors contributed to the evolutionary history of AS in vertebrates remains largely unclear. Recently, a study using millions of synthetic mini-genes with degenerated subsequences demonstrated that the likelihood of AS decreases exponentially with increasing distance between constitutive and newly introduced alternative SS (Rosenberg et al., 2015), suggesting that sequence changes between constitutive and alternative SS might contribute to the changes of AS among species. In plants, however, the detailed mechanisms that affect AS remain largely unclear (Reddy et al., 2013). Although it has been proposed that changes in chromatin features such as DNA methylation, histone marks as well as RNA structural features, and SREs are important in regulating AS in plants, experimental evidence is largely lacking (Reddy et al., 2013). A recent study shows that DNA methylation could affect AS in rice (Wang X.T. et al., 2016), indicating changes in DNA methylation can contribute to the variations of AS among species, however, this hypothesis has not been thoroughly tested.

Because AS regulation is a complex process involving many factors, computational modeling is a useful tool to identify key factors and predict the outcome of splicing. While the Bayesian neural network (BNN) method was developed for decoding the splicing code in mammals (Barbosa-Morais et al., 2012), deep learning approaches, which refers to methods that map data through multiple levels of abstraction, have recently been shown to surpass BNN-based approaches (Leung et al., 2014; Mamoshina et al., 2016). Furthermore, deep learning methods are also able to cope with large, heterogeneous and high-dimensional datasets, an issue that is involved in predicting DNA and RBPs (Alipanahi et al., 2015) and AS (Leung et al., 2014; Mamoshina et al., 2016).

Here, we performed a comparative analysis of the transcriptomes of both closely and distantly related plant species to explore the evolutionary history of AS in plants. To further understand the mechanisms underlying the AS evolution in plants, we applied a deep learning approach to investigate the determinants of AS and their effects on AS evolution. Specifically, we aimed to address the following questions in plants: (1) What are the evolutionary patterns of AS? (2) Are the AS events that are coupled with NMD more conserved than regular AS events? (3) What are the important AS determinants? (4) Which AS determinants contributed to the AS divergence between closely related plant species?

## Materials and Methods

### Read Mapping, Transcripts Assembly, and Abundance Estimation

All RNA-seq data of *Nicotiana attenuata* were generated in our lab, while the data of other species were downloaded from the short reads archive ^1^. The mapping information and SRA IDs of all datasets are listed in Supplementary Tables S1, S2. All of the RNA-seq reads were generated from polyA selected libraries. The raw sequence reads were trimmed using AdapterRemoval (v1.1) (Lindgreen, 2012) with parameters “–collapse –trimns –trimqualities 2 –minlength 36.” The trimmed reads from each species were then aligned to the respective reference genome using Tophat2 (v2.0.6) (Trapnell et al., 2009), with maximum and minimum intron size set to 50,000 and 41 bp, respectively. After our analysis, we noticed that introns in plants are usually larger than 60 bp. However, in our dataset, only less than 0.2% of introns are less than 60 bp. Therefore, including these small introns (between 41 and 60 bp) that might be due to mapping errors should not affect the results. The numbers of uniquely mapped reads and splice junctions mapped reads were then counted using SAMtools (v0.1.19) (Li et al., 2009) by searching “50” in the MAPQ string and “^∗^N^∗^” flag in the CIGAR string of the resulting BAM files. The uniquely mapped reads from each sample were sub-sampled with the same sequencing depth (17 million) using SAMtools (v0.1.19) (Li et al., 2009).

The transcripts of each species were assembled using Cufflinks (v2.2.0) (Trapnell et al., 2012) with the genome annotation as the reference. The open reading frame (ORF) of each transcript was analyzed using TransDecoder from TRINITY (v2.1.0) (Grabherr et al., 2011). To estimate the expression level of genes/transcripts, all trimmed reads were re-mapped to the assembled transcripts using RSEM (v1.2.8) (Grabherr et al., 2011). Transcripts per million (TPM) was calculated for each gene/transcript (Wagner et al., 2012). Only genes with TPM greater than five in at least one sample were considered as an expressed gene.

### AS Detection

All AS analysis were based on splicing junctions obtained from the BAM files produced by Tophat2. To remove the false positive junctions that were likely due to non-specific or erroneous alignments, all original junctions were removed if the overhang size was smaller than 13 bp, as suggested in Ling et al. (2015). All filtered junctions were then used for AS identification and annotation using JUNCBASE v0.6 (Graveley et al., 2011). Due to the relatively low sequencing depth of each individual sample of Brassicaceae RNA-seq data (Supplementary Table S2), we merged the BAM files of each three replicates together and randomly subsampled 17 million (the lowest depth among all merged samples) unique mapped reads from each merged file to avoid the heterogeneity of sequencing depth. The summary of all detected junctions is shown in Supplementary Table S3.

The percent spliced index (PSI) of each AS event, which represents the relative ratio of two different isoforms generated by the AS was calculated in each sample. PSI = (number of reads of inclusion isoform)/(number of reads of inclusion isoform + number of reads to exclusion isoform) as suggested in Graveley et al. (2011), Lareau and Brenner (2015). To avoid false-positives, only AS events that supported by at least 10 reads were considered. For alternative 5’ donor site (AltD), alternative 5’ acceptor site (AltA), and exon skipping (ES), the number of supporting reads was calculated as the sum of reads that support junctions, whereas for intron retention (IR), the total number of supporting reads was calculated as the sum of reads that mapped to both junctions and the intron region.

### Identification of Conserved Exon–Exon Junctions (EEJs) and AS

We separately extracted the 100 bp sequence from the flanking upstream exon and downstream exon of each junction that has mapped read to support, and combine each side of exon sequence (in total 200 bp sequence) to represent the EEJ. The sequences of all EEJs were compared between species using TBLASTX (v.2.2.25) (Altschul et al., 1990) to find homologous relationships (Supplementary Figure S1). A python script was used to filter the TBLASTX results based on the following requirements: (1) The gene pair containing the EEJs must be the one-to-one orthologous gene pair between the two species; (2) the EEJ sequences between two species must be the best reciprocal blast hit based on the bit score; (3) at least 3 amino acid (aa) from both the flanking upstream exon and downstream exon sequence were aligned and (4) alignment coverage > = 60 bp, (5) *E*-value < 1E-3.

We only consider an AS event to be conserved if the same type of AS was found on the conserved EEJs between two plant species.

### Identification of AS Events That Generate Premature Termination Codons (PTC)

The junctions related to each AS event were mapped back to assembled transcripts; only AS events which were related to junctions that mapped to two unique transcripts (had no structural difference except the AS region) were retained to avoid the situation where the sequence differences of the two transcripts resulted from multiple AS events. The transcript was considered to have a PTC if the stop codon of the longest ORF was at least 50 nucleotides upstream of an exon–exon boundary (the 50 nucleotides rule) (Nagy and Maquat, 1998; Schoenberg and Maquat, 2012; Weischenfeldt et al., 2012). To identify AS events that generate PTC-containing and non-PTC-containing transcripts, we used following criteria: (a) the AS events that can only be mapped to two unique transcripts; (b) the AS region is the only difference between the two transcripts; (c) at least one transcript does not contain PTC, as the AS events that generate two PTC-containing transcripts are likely due to assembly or annotation errors.

### One-to-One Orthologous Gene Identifications and Gene Family Size Estimation

One-to-one orthologous gene pairs were predicted based on pair-wise sequence similarities between species of the corresponding dataset. First, we calculated the sequence similarities between all protein-coding genes using BLASTP for the selected species and filtered the results based on *E*-value less than 1E-6. Second, we selected the groups of genes that represent the best reciprocal hits that are shared among all species from the corresponding dataset.

For calculating the gene family size, we first defined gene families among different species by using a similarity-based approach. To do so, the homolog groups that were identified from our previous work were used, which were predicted from 11 plant species (Xu et al., 2017). In brief, all-vs.-all BLASTP was used to compare the sequence similarity of all protein coding genes, and the results were filtered based on the following criteria: *E*-value less than 1E-20; match length greater than 60 amino acids; sequence coverage greater than 60% and identity greater than 50%. All BLASTP results that remained after filtering were clustered into gene families using the Markov cluster algorithm (mcl). The gene family size for a species is represented by the number of genes of this species within the corresponding gene family.

### Correlation and Clustering

For the pairwise comparison of AS, Spearman correlation and binary distance was applied to the PSI data (0.05 < PSI < 0.95 in at least one sample) and binary data (only one-to-one orthologous were used, and all genes that had no AS in all of the four species were excluded), respectively. A non-parametric correlation was selected for PSI level because of its bimodal nature distribution (0 and 100). For the pairwise comparison of gene expression, Pearson correlation was applied to log_2_ (TPM+1) of expressed genes to avoid infinite values.

The R package “pvcluster” was used for clustering of samples with 1,000 bootstrap replications. When we clustered and performed principal component analysis (PCA) of gene expression, the TPM values were normalized by GC% (EDASeq package in R) and TMM (the trimmed mean of *M*-values).

### Identification of Possible Alternative Splice Sites (SS) and Regulatory Sequences

The 5′ and 3′ SS including 5 bp up and downstream sequences of all EEJs were used as the positive dataset, while the sequences extracted using the same method for all inter-GT (for 5′ splice site) and inter-AG (for 3′ splice site) within junction regions were used as background dataset. The putative SS motifs (12-mer) of both 5′ and 3′ SS were separately identified using Homer V3.12 (Heinz et al., 2010) and only motifs present in at least 5% of total positive sequences and *P*-value < 1E-20 were kept. The appearance of putative SS was identified using scanMotifGenomeWide, a Perl script included in the Homer toolkits and only sequence regions with match score > 2 were kept.

Homer was also used to identify the putative regulatory intronic and exonic sequence motifs (6-mer) of AltD, AltA and IR. The 50 bp up and downstream sequence of 5′ SS was regarded as exonic and intronic sequence and vice versa for 3′ SS. For AltD and AltA, the related sequences of EEJs with AS were used as the positive dataset, while 10,000 related sequences of EEJs without AS by random selection (due to a large number of sequences) were used as background dataset. The enriched motifs in the positive dataset were regarded as splicing enhancers, while the enriched motifs in the negative dataset were considered as splicing silencers. For IR, the related sequences from both SS of EEJs with IR were used as the positive dataset and the same sequences from EEJs without IR were used as background dataset. The conserved motifs between species were identified using compareMotifs, a Perl script included in the Homer toolkits and only one mismatch was allowed. To identify polypyrimidine tracts, UA-rich tracts and branch site of each EEJ, we used the algorithm and scripts from Schwartz et al. (2008) and Szcześniak et al. (2013). In brief, polypyrimidine tracts and UA-rich tracts, intronic regions of up to 50 bases upstream of the 3′ SS were searched using the algorithm that searches for the longest string with the C + U (in the case of polypyrimidine tracts) or A + U (for UA tracts) composition exceeding 85%. Polypyrimidine tracts that end within the last 10 bases of an intron were considered. Moreover, the tracts were required to be at least five bases long. The branch site identification consists three steps. First, the 100 nucleotides (nt) upstream of the 3′ SS were used to identify the heptamers that were found in other systems (Bon et al., 2003): NNYTRAY, NNCTYAC, NNRTAAC, and NNCTAAA, Second, each heptamer was scored according to the number of mismatches from the optimal consensus of TACTAAC. Third, branch site containing introns were considered only if the introns in which the most downstream hit also has the best score. Although the last step discarded a relatively large fraction of introns, it reduces the false-positive rate significantly (Schwartz et al., 2008). To estimate the effect of each putative sequence motif, polypyrimidine and UA-tracts, we calculated the AS frequency of EEJs containing or not containing the motif/tract. Then for each motif/tract, the log_2_ odds ratio (effect size) with and without the motif/tract were calculated to quantify to what extent the presence of the motif/tract increases or decreases the AS frequency compare to its absence:

$$\mathrm{Effect Size}=\log2\frac{p(\mathrm{AS}|\mathrm{motif})/(1-p(\mathrm{AS}|\mathrm{motif}))}{p(\mathrm{AS}|-\mathrm{motif})/(1-p(\mathrm{AS}|-\mathrm{motif}))}$$

### Deciphering the Splicing Codes and AS Conservation Using a Deep Learning Algorithm

To investigate which sequence determinants contributed to the AS in plants, we constructed multi-layer feed-forward artificial neural networks using H_2_O’s deep learning algorithm (“h2o” package) in R 3.0.2 (R Development Core Team 2013). For each AS type, a matrix was created based on the information of all EEJs that contain the AS (only that single event) and other EEJs within the same gene. The AS status (either AS or constitutive) was considered as output and the features that were known to be associated with splicing recognition and regulation in eukaryotes (Lewandowska et al., 2004; Kandul and Noor, 2009; Rosenberg et al., 2015) (listed in Supplementary Data Sheet S1) were used as input for training the model. On average, 25 features were used in the AltA model and 13 features were used in the AltD model. “TanhWithDropout” was used as the activation function and three hidden layers were used. Furthermore, “logloss” was used for model selection and the “Gedeon” method was used to compute the variable importances for input features. To reduce the background noise, we removed the EEJs which were supported by less than five reads on average. In addition, because the number of constitutively spliced EEJs in all cases is much larger than alternatively spliced EEJs, we randomly selected the same number of constitutive spliced EEJs as negative examples and combined them together with all alternative spliced EEJs as the full dataset (50% precision by chance). To train and test the deep neural networks (DNN), the full dataset was randomly split, which 60% of data were used for training, 20% used for validation and the other 20% were reserved for testing. We trained for a fixed number (10,000) of epochs or stopped the training once the top 10 model were within 1% of improvement, and selected the hyper-parameters that gave the optimal area under the receiver operating characteristic curve (AUC) performance on the validation data. The model was then retrained using these selected hyper-parameters with the full dataset. The AUC value (range between 0 and 1) is an indicator showing the performance of a classification model, which is equivalent to the probability that a randomly chosen positive example is ranked higher than a randomly chosen negative example. A higher AUC value indicates a better performance of the model (high precision and high specificity).

Using a similar approach, we constructed the model for AS conservation. For each AS type, a matrix was created based on the information of all orthologous EEJ pairs between two species that contain the AS in at least one species. To reduce the background noise, any EEJ with multiple AS types, low number of support reads (less than five) or orthologous EEJ pair have different AS types were removed. The conservation levels (conserved, lost or gained in the other species) were used as the output of the model and the difference of features that were known to be important to AS and AS conservation (Su et al., 2006; Kelley et al., 2014; Sierro et al., 2014; Lambert et al., 2015; Rosenberg et al., 2015) (listed in Supplementary Data Sheet S2) between two species were used as input to train the model. Yass v1.15 was used to align the SS’ flanking sequences (combined 50 bp upstream and downstream sequences of 5′/3′ splice site, 100 bp in total) of each orthologous EEJ pair, the similarity was calculated as: (length of alignment – number of gaps – number of mismatches)/(total sequence length). To reduce the bias from different transition types in the dataset (much higher proportion of loss/gain than conserved AS), the data used to train the model was selected as the ratio of 1:1:1 for conserved, lost and gained situations (33.3% precision by chance). Due to small sample size of conserved AS, the model based on the same original data may differ as the randomly selected data of AS lost/gained were different each time. Therefore, the model construction process was repeated 10 times and the models that achieved the highest AUC for the complete dataset were considered. The one-to-one ortholog gene list, genes with/without AS from the four eudicot species are provided in Supplementary Data Sheet S3.

## Results

### Genome-Wide AS Patterns Are Species-Specific in Plants

To provide an overview of AS evolution among different plant families, we studied the genome-wide AS in *A. thaliana*, soybean (*Glycine max*), tomato (*Solanum lycopersicum*), and wild tobacco (*N. attenuata*), from which comparable transcriptomic datasets are available from the same tissues (roots, leaves, and flowers) and they represent a wide-range of eudicots. The overall distributions of different AS types within each species are consistent with previous studies. In all investigated species, intron retention (IR) and alternative 3′ acceptor site (AltA) are the two major AS types (Supplementary Figure S2; Aoki et al., 2010; Marquez et al., 2012; Shen et al., 2014; Ling et al., 2015).

**FIGURE 1:**
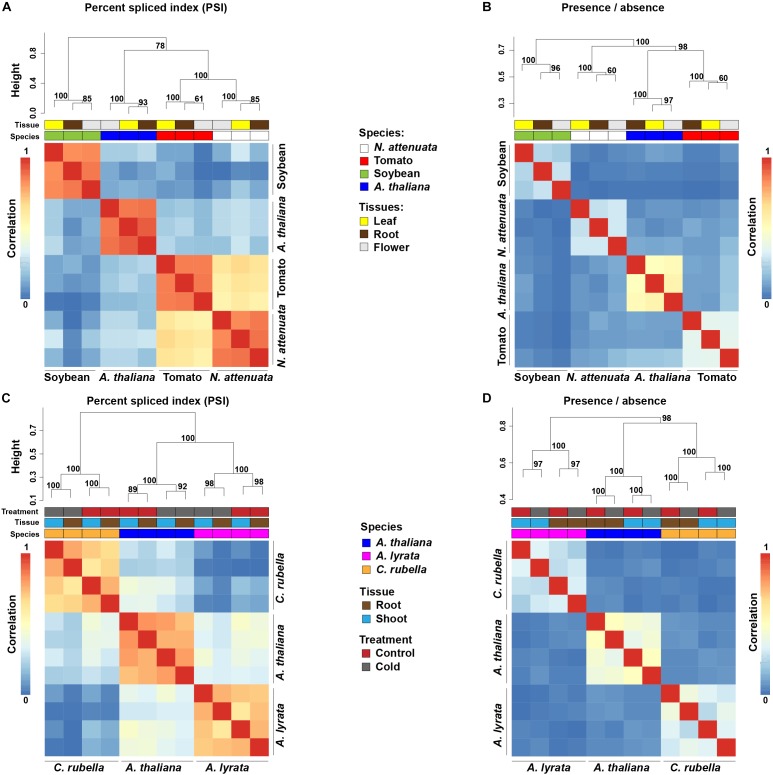
Species-specific clustering of alternative splicing (AS) among different plant species. **(A,C)** Heatmaps depict species-specific clustering based on PSI values among four eudicots species **(A)** and three Brassicaceae species **(C)**. The clustering is based on conserved splicing junctions (**A,C**: *n* = 502 and 5241, respectively). **(B,D)** Heatmaps depict species-specific clustering based on presence and absence of AS of the one-to-one orthologous genes. In total, junctions from 3,857 **(B)** and 6,262 **(D)** orthologous were used for the clustering. Numbers at each branch node represent the approximately unbiased bootstrap value calculated from 1,000 bootstrap replications. The color code above each heatmap represents species, tissue, and treatments.

To investigate the evolutionary patterns of AS, we compared AS profiles across selected tissues and species. Because sequencing depth is known to strongly affect AS detection, we randomly subsampled 17 million (the lowest depth among all samples) uniquely mapped reads from each sample to standardize for the heterogeneity of sequencing depths. Overall, more than 75% of the splice junctions that were identified from the full dataset can be detected from these randomly selected 17 million uniquely mapped (later referred as 17M) reads (Supplementary Figure S3A), indicating the 17M reads is sufficient to reveal the AS evolution pattern among species. In addition, plotting the saturation curve of detected splice junctions with different sequencing depths showed that 17M reads have reached or at least are close to the saturation point (Supplementary Figure S3B). Thus, all downstream comparative analyses were based on this subsampled dataset. To investigate the conservation level of AS among different plant species, we focused on only one-to-one orthologous relationships, because relationships among complex one-to-many or many-to-many orthologous relationships are much more difficult to infer. Clustering analyses using PSI (Graveley et al., 2011; Lareau and Brenner, 2015) that measures the quantitative differences of AS among samples showed that different tissues of the same species are more similar to each other than the same tissue from different species (Figure 1A). Using the measures of AS that consider the presence or absence of AS (binary) from the genes that are one-to-one orthologous among all compared species, the same species-specific clustering pattern was found (Figure 1B). Consistent results were also obtained using all available reads (Supplementary Figures S3C,D) or when each type of AS was analyzed separately (Supplementary Figure S4).

To further investigate the evolutionary patterns of AS among closely related species, we analyzed a recently published transcriptome dataset from three Brassicaceae species (*A. thaliana*, *A. lyrata*, and *Capsella rubella*), each of which ha comparable transcriptome data from two tissues (root and shoot) and two treatments (control and cold treated). Using both quantitative (PSI) and qualitative measures (binary) of AS, a similar species-specific clustering pattern was observed (Figure 1C,D). Interestingly, within same species and same tissue, samples exposed to cold stress clustered together regarding levels of PSI (Figure 1C), a result which is consistent with previous studies that demonstrate that stresses can induce genome-wide AS responses (Li et al., 2013; Ding et al., 2014; Ling et al., 2015).

Species-specific clustering patterns were also reported at the level of GE of one-to-one orthologs among *A. thaliana*, rice and maize (Yang and Wang, 2013). To examine whether species-specific AS clustering results from GE divergences, we compared the divergence patterns of AS and GE among transcriptomes of different species. Comparisons among species from different plant families showed that both GE and AS cluster in species-specific patterns (Figure 1A,B and Supplementary Figures S5A,B). However, when species from the same plant family are compared, such as tomato and *N. attenuata* (Solanaceae), the species-specific AS pattern remained (Figure 1A,B), but the GE data clustered in a tissues-specific pattern (Supplementary Figures S5A,B). This shows that the expression profiles of the same tissues from different species are more similar to each other than the expression patterns from different tissues of the same species, indicating that the observed species-specific AS clustering is not due to GE divergence. A similar pattern was also found in the expression profiles of tissue samples from the three Brassicaceae species, among which the expression profiles of shoots and roots from different species were clearly separated (Supplementary Figures S5C,D). The observed difference in species-specific clustering patterns between GE and AS is consistent with the pattern found in animals (Barbosa-Morais et al., 2012; Merkin et al., 2012).

### Massive Gains and Losses of AS Among Different Species

Species-specific clustering of AS pattern suggests a low level of AS conservation among species. Overall, among 3,857 one-to-one orthologous genes among the four eudicot species that have AS in at least one species, only ∼7% of them have AS in all four species, while ∼41% of them have species-specific AS. A similar pattern was also found when using the full dataset (not subsampled). We further investigated the pattern by looking at each exon–exon junction (EEJ) among orthologous groups, and found that more than 87.7% of AS events were species-specific (Supplementary Figure S6). Because the rapid change of AS could result from the rapid loss or gain of EEJ between species, we further compared the conservation of EEJs and AS among orthologous genes. Among the four eudicot species, 60% of EEJs are conserved in at least two species, which is much higher than the conservation of AS (∼12%). Additional analysis showed that 92% of AS events identified from the conserved EEJs (shared among all four species) are species-specific. A similar analysis using the data from the three Brassicaceae species revealed the similar pattern (Supplementary Figures S7A,B). Together, the results from the comparison between divergent species and closely related species consistently suggest that AS are highly variable among plants.

**FIGURE 2:**
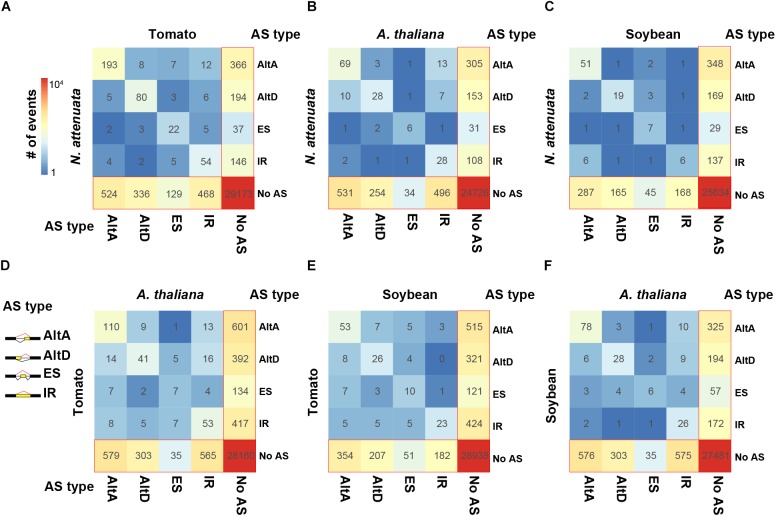
Transition spectrum of AS between each species pairs. **(A)** *N. attenuata* vs. tomato, **(B)** *N. attenuata* vs. *A. thaliana*, **(C)** *N. attenuata* vs. soybean, **(D)** tomato vs. *A. thaliana*, **(E)** tomato vs. soybean, and **(F)** soybean vs. *A. thaliana*. The color of each grid indicates the abundance of the transitions between two AS types. The values are also shown. Only exon–exon junctions (EEJs) with at most one AS event were considered. AltA: alternative 3′ acceptor site; AltD: alternative 5′ donor site; ES, exon skipping; IR, intron retention.

To investigate the transition spectrum of AS at the conserved EEJs between species pairs, we calculated the AS changes among different types of AS. Among the four eudicot species, while the transitions among different AS types are rare, the gain/loss of AS is the most abundant transition type among all three pairwise comparisons (Figure 2A–F). For example, while an AltA event was found in *XCT* in *N. attenuata*, which was also confirmed by RT-PCR in our previous work (Ling et al., 2015), no AS was found at its orthologous junction in tomato (Supplementary Figure S8D). Among different AS types, AltA and exon skipping (ES) are the most and least conserved AS, respectively. Similar patterns were observed among three closely related species in Brassicaceae (Supplementary Figures S8A–C). These results suggest that the species-specific AS pattern is largely not due to the changes of EEJs among species, but rather the species-specific gains and losses of AS.

### The AS Events That Result in PTC-Containing Transcripts Are Likely More Conserved Than Others

**FIGURE 3:**
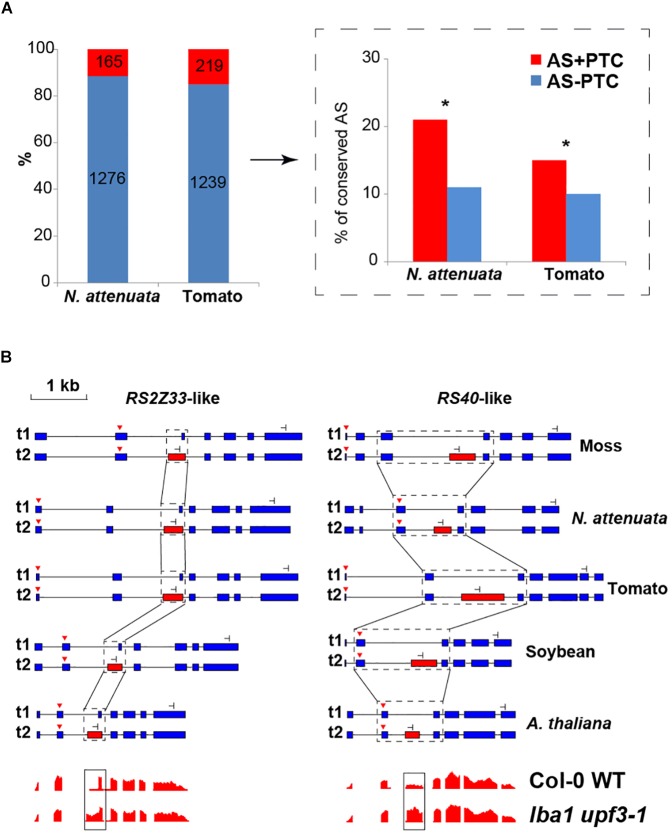
The conservation of AS between AS+PTC and AS-PTC. **(A)** The number and relative portions of AS±PTC in *N. attenuata* and tomato. The insert indicated by the black arrow depicts the percentage of AS+PTC and AS-PTC that are conserved between *N. attenuata* and tomato. Asterisks indicate the significant difference between two AS groups as determined by Fisher’s exact test (*P* < 0.05). **(B)** Conserved AS between moss and eudicots in serine/arginine-rich splicing factor *RS2Z33*-like and *RS40*-like gene. The diagrams of the structure of transcripts generated by the AS in all five species, the dominant and minor transcripts are represented by t1 and t2, respectively. Constitutive exons are represented by blue boxes, alternatively spliced exons are represented by red boxes and introns are represented by black solid lines. The black dotted boxes highlight the conserved AS and the start and stop codons are shown as red triangles and stop signs, respectively. The diagrams in the bottom panel showed the relative reads coverage of *AtRS2Z33* and *AtRS40* exons in wild-type plant and *lba1 upf3-1* double mutants. The black box highlights the coverage of the spliced region which is significantly increased in *lba1 upf3-1* double mutants (The diagrams are modified based on the data shown in http://gbrowse.cbio.mskcc.org/gb/gbrowse/NMD201).

Previous studies suggest that many pre-mRNAs underwent unproductive AS, which generates transcripts with in-frame PTCs that are coupled with NMD in plants (Schwartz et al., 2006; Hori and Watanabe, 2007; Kerenyi et al., 2008;Kalyna et al., 2012; Drechsel et al., 2013). To investigate whether unproductive AS can affect the AS conservation and contribute to the loss/gain of AS among different plant species, we separated the AS into two groups: (1) AS+ PTC and (2) AS- PTC (details see section “Materials and Methods”). Overall, the portion of AS+ PTC ranges from 9 to 15% among the four dicots (Supplementary Figure S9), suggesting that only a small portion of AS generated PTC-containing transcripts. Comparing the levels of conservation between tomato and *N. attenuata*, we found the AS+PTC is significantly more conserved than AS-PTC (*P* < 0.02, Figure 3A). For example, among nine AS+PTC of *N. attenuata* which are both conserved and have PTC information in tomato, eight of them (89%) also generated +PTC transcripts in tomato. Similar patterns were also observed in the three Brassica species (Supplementary Figures S10A,B).

To further investigate the level of conservation of AS+PTC, we extended our analysis by adding the transcriptome data of a very ancient plant species, the spreading earthmoss (*Physcomitrella patens*). Our rationale is that if AS+PTC events are more conserved than AS-PTC events, we would expect to see many AS+PTC events from the ultra-conserved AS events. Here, we focused on the 10 most highly conserved AS events found in all four eudicot plants (Supplementary Figure S6B) and checked for their presence in moss. In total, we found six AS events that were also present in moss, indicating these AS events might have evolved since land plants and played essential functions in plants. Interestingly, two of these ultra-conserved AS events were from serine/arginine-rich (SR) genes (*RS2Z33*-like and *RS40*-like), which are part of the RNA splicing machinery. The *RS2Z33*-like gene also has AS in rice and *Pinus taeda* (Iida and Go, 2006; Kalyna et al., 2006). Analyzing the protein coding potential of the transcripts generated by these six ultra-conserved AS events showed that five resulted in +PTC transcripts. For example, the AS events of *RS2Z33*-like and *RS40*-like genes result in +PTC alternative transcripts in all five species and are likely the targets of NMD (Figure 3B). To further investigate whether these +PTC transcripts are affected by NMD, we analyzed the available transcriptome data from *A. thaliana* wild-type (WT) and NMD-deficient (*lba1* and *upf3-1* double mutant) plants (Drechsel et al., 2013). Among all five +PTC transcripts in *A. thaliana*, three showed significantly higher expression in NMD-deficient plants (*P* < 7e-06), including *RS2Z33*-like and *RS40*-like genes (Figure 3B). Together, these results suggest that AS coupled with PTC is likely more conserved than regular AS and some of these AS+PTC pairs may play essential roles in plants.

### Mechanisms Involved in Determining AS Are Overall Conserved Among Different Plant Species

To further understand the mechanisms that contribute to the divergence of AS among species, it is necessary to identify the key features of AS in plants, which are largely unknown (Reddy et al., 2013; Staiger and Brown, 2013). Because splicing is often mediated by SS, we first investigated whether the SS were different between constitutively and alternatively spliced junctions. Comparisons of the SS and their surrounding 12 bp sequences between constitutively and alternatively spliced junctions revealed that their SS are overall very similar (Supplementary Figure S11). Furthermore, we separately identified sequence motifs (12-mer) that are enriched in 5′ and 3′ SS compared to random sequences and found that these identified motifs are also highly conserved among the studied species (Supplementary Figure S12).

**FIGURE 4:**
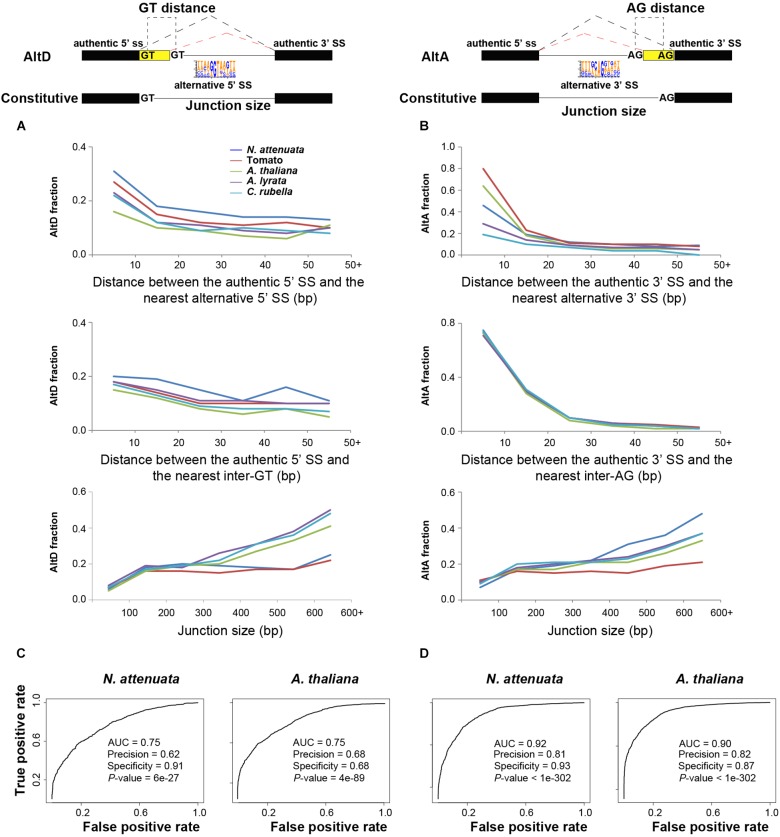
The determinants of alternative 5′ donor site (AltD) and alternative 3′ acceptor site (AltA) in plants. **(A,B)** The frequencies of AltD/AltA on junctions with different distance between the authentic splice site (SS) and the nearest alternative SS (5′ ss and 3′ ss, respectively), and the distance between authentic SS and the nearest inter GT/AG and junction size. **(C,D)** The area under the curve (AUC) plot of deep learning models using the key determinants of AltD and AltA in *N. attenuata* and *A. thaliana*. The model performance including AUC, accuracy, specificity and significance are also shown.

From the mechanistic point of view, the junction size (distance between the 3′ SS and 5′ SS of the two exons), the presence and positions of alternative SS, which are the additional SS motifs that compete with the authentic splice donors or acceptors can be important for the regulations of different types of AS (Gopal et al., 2005; Kandul and Noor, 2009; Braunschweig et al., 2014; Rosenberg et al., 2015). For the different AS types, we compared these features from both constitutively and alternatively spliced junctions. Because ES events are rare in all species, we only studied the three most abundant AS types AltD, AltA, and IR. As expected, the results showed that for a given junction, the likelihood of both AltD and AltA increases with junction size, while the likelihood of both AltD and AltA decreases with the distance between authentic and alternative SS as well as the distance between authentic SS and the nearest internal GT/AG (Figure 4A,B). Interestingly, although the likelihood of IR in smaller junctions appears larger than in large junctions, no significant correlation with junction size was found (Supplementary Figure S13A). Both 5′ and 3′ SS of the junction with IR are significantly weaker than those of the constitutive junction (Supplementary Figure S13B).

Furthermore, the presence/absence of UA-rich tract, polypyrimidine tract and branch site are also known to be associated with 3′ SS recognition in eukaryotes (Lewandowska et al., 2004; Fu and Ares, 2014). We compared the frequency of AltA and IR between junctions of the AS gene with and without the presence of UA, polypyrimidine tract and branch site within 100 bp upstream of 3′ SS. We found that the frequencies of both AltA and IR are significantly higher in the junctions without UA and polypyrimidine tract than the junctions with them, while the presence of branch site had no significant effect (Supplementary Figure S14).

*Cis*-regulatory elements, including splicing enhancers and silencers located close to SS are also important for the regulations of splicing. To identify these candidate regulatory elements, we performed a *de novo* hexamer motif enrichment analysis by comparing 50 bp sequences from the 5′ and 3′ sides of both donor and acceptor sites between alternatively spliced and constitutively spliced junctions. The results showed that most of the putative enhancer motifs for alternatively spliced junctions are highly similar to the identified SS. In addition, we also identified several putative silencer motifs (range from 5 to 10 for AltD and 10 to 18 for AltA in the five species), some of which were significantly more enriched in constitutively spliced junctions than alternatively spliced junctions in all species (Supplementary Figures S15A,B). However, it is worth noticing that the *cis*-regulatory elements that are located 50 bp further away from the SS or less than 6 bp may have been missed from our analysis.

To evaluate whether these identified features represent the AS determinants, we used a machine learning approach and modeled the different types of AS in each of the studied species. The rationale for this approach is that if the features we identified as representative of the key AS determinants were accurate, we would be able to predict whether an exon-intron junction is constitutively or alternatively spliced based on their quantitative or qualitative information. For this, we combined all of the extracted featured mentioned above. In addition, we also extracted information on whether the alternative SS would introduce a frameshift, which may result in premature terminate code (PTC) or different open reading frames (ORFs), the number of reads that support the junction, which represent levels of expression that is known to be associated with AS, as well as the presence and absence of the identified *cis*-motifs. Using this information, our model achieved high precision and specificity for both AltD and AltA in all five species (Figure 4C,D and Supplementary Figures S16A,B), which suggests that the identified features can provide sufficient information to discriminate AS junctions from constitutively spliced junctions. However, for IR, the extracted features were not predictively useful, as the average model performance measured by area under the receiver operating characteristic curve (AUC) was only 0.54, suggesting low precision and low specificity. This indicates that additional undetected factors have contributed to the determination of IR.

This modeling approach further provides indicative information on the relative importance of each feature to the prediction model. The results showed that for AltD, the distance between the authentic SS and the nearest alternative 5′ SS or inter GT, the junction size and presence/absence of 5′ additional SS in the intron are among the top important features for the prediction in all species (Supplementary Data Sheet S1). In addition, the frame shifts introduced by the nearest alternative 5′ SS and nearest GT were also important contributors to the model (Supplementary Data Sheet S1). For AltA, the distance to the nearest inter-AG dinucleotide is the top feature for the prediction among all five species. Interestingly, all of the identified putative silencers/enhancers (6-mers motifs) only had a marginal role for the predictions of both AltD and AltA (Supplementary Data Sheet S1), the same top important features were presented in models without these motif features. Together, these results showed that the mechanisms regulating AltD and AltA are likely overall conserved among the studied species.

### Changes in AS Determinants Contributed to the Divergence of AS in Plants

The relatively conserved AS regulation mechanisms among studied species provide a foundation for investigating the mechanisms that contributed to the divergence of AS among closely related plant species. We hypothesized that the changes in the identified AS determinants among species resulted in the divergence of AS among species. To test this, we associated the changes of the identified AS determinants and AS conservation among closely related species. Because we did not find determinants for IR, we only focused on the evolution of AltA and AltD.

**FIGURE 5:**
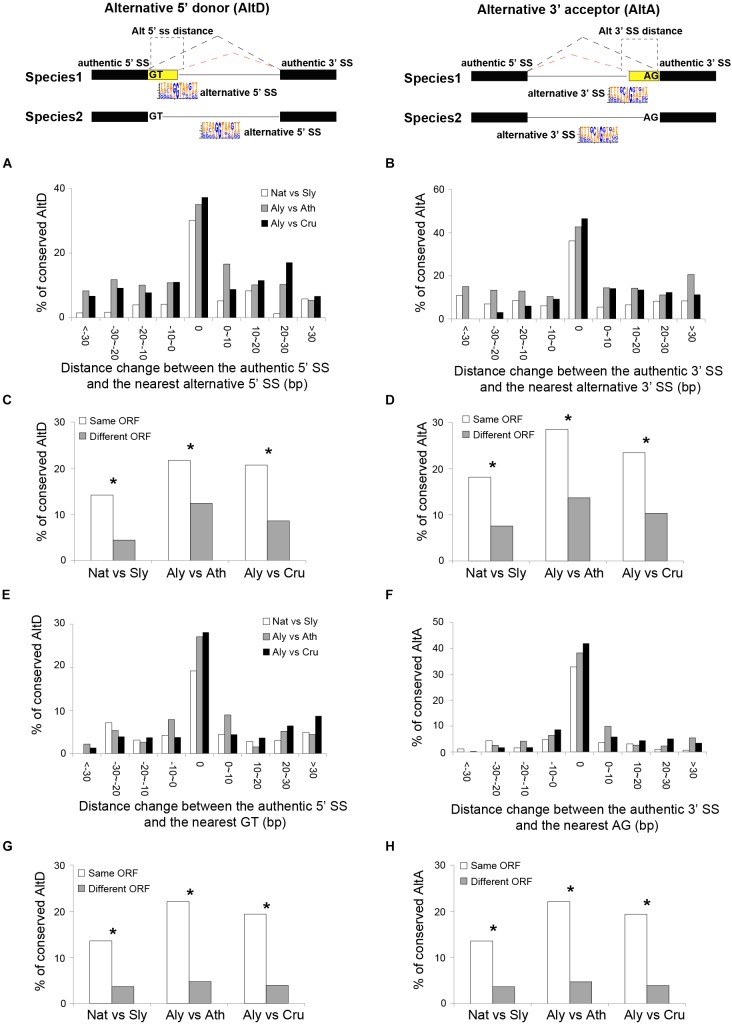
Features that affect the conservation of AltD and AltA between closely related plant species. **(A,B)** The portion of conserved AltD/AltA decreases with the distance between authentic and alternative splice site (SS) between two species. **(C,D)** The percentage of conserved AltD/AltA which the nearest alternative 5′/3′ SS generates transcripts with same or different open reading frame (ORF) between species. **(E,F)** The portion of conserved AltD/AltA decreases with changes in the distance between authentic SS and nearest inter-GT/AG sites between two species. **(G,H)** The percentage of conserved AltD/AltA in which that the nearest inter-GT/AG generates transcripts with same or different ORF between species. Nat, *N. attenuata*; Sly, Tomato; Ath, *A. thaliana*; Aly, *A. lyrata*. The asterisks indicate the significance as determined by Fisher’s exact test (*P* < 0.05).

Variation in the distance between authentic SS and alternative SS or inter-GT/AG were negatively associated with AS conservation: the levels of AS conservation decreased with increasing distance in all three pairs of comparisons (Figure 5A,B), for both AltD and AltA. In addition, the changes in the reading frame introduced by the alternative SS also significantly decreased the conservations of both AltA and AltD (Figure 5C,D). The similar pattern was also found for the distance between authentic SS and the nearest inter-GT/AG (Figure 5E–H).

Variation in the *cis*-regulatory elements UA-tract, polypyrimidine tract and branch site significantly reduced the conservation for AltA, but did not affect the conservation of AltD among species (Supplementary Figures S17A,B and Supplementary Data Sheet S2). This result is consistent with the functional roles of these *cis*-regulatory elements in regulating AltA.

To further systematically analyze different factors that might affect the conservation of AS, we constructed an AS evolution model for each closely related species pair using a deep learning method. In addition to the key AS determinants identified in this study, we also included several other features that were previously hypothesized to be important for AS conservation between species in the model, such as changes in copy numbers (role of gene duplications), transposable element (TE) insertion within the junction, GC-content and sequence similarity of SS. For the AltD, all three models between species pairs achieved significantly better prediction than by chance (highest *P*-value = 3e-44), with an average precision of 0.63 and specificity of 0.82. In all three pairwise comparison models, the distance changes between authentic and nearest alternative 5′ SS or inter-GT/AG are among the top five important features (Supplementary Figure S18A and Supplementary Data Sheet S2). For AltA, all three models achieved a precision and specificity (average 0.70 and 0.85, respectively) that was significantly higher than by chance (highest *P*-value = 3e-145). In all three models, distance changes between authentic SS and the nearest inter-AG or alternative 3′ SS and the changes on *cis*-regulatory elements (UA and polypyrimidine tracts) represent the top five most important features that contributed to the model predictions (Supplementary Figure S18B and Supplementary Data Sheet S2).

Interestingly, we found TE insertions to also be an important factor that reduced the conservation of both AltD and AltA between *N. attenuata* and tomato but not between any pair of the Brassicaceae species (Supplementary Figures S18A,B). This is likely due to the difference of TE abundance between *N. attenuata* (∼63%) and tomato (∼81%), values which are much higher than the differences between *A. thaliana* (∼23%) and *A. lyrata* (28%) (Hu et al., 2011; Tomato Genome Consortium, 2012). Furthermore, we also analyzed the impact of DNA methylation changes between *A. thaliana* and *A. lyrata* using data from (Seymour et al., 2014) and found no significant effects (Supplementary Figures S18A,B).

## Discussion

Here, we showed that species-specific gain and loss of AS resulted in lineage-specific AS profiles in plants. Between closely related species, AS events that introduce PTCs are likely more conserved than AS events that do not introduce PTC (Figure 3A). Consistently, several AS events that generate PTC-containing transcripts were ultra-conserved among highly divergent plants. To understand the mechanisms that resulted in a rapid divergence of AS between closely related species, we identified several key determinants for both alternative donor (AltD) and alternative acceptor (AltA) splicing. We found that the change of these key determinants between species is associated with the gain and loss of AS in plants.

In this analysis, we observed a dominant species-specific pattern of AS among different species (Figure 1). Although, the relatively low sequence depth (17 million) or incomplete genome assembly and annotation might be a confounding effect to draw this conclusion. We did several analyses to examine this and found: (i) overall, 17M unique mapped reads can sufficiently detect more than 75% of total splice junctions in all four species (Supplementary Figure S3A); (ii) increase of sequencing depth from 17M didn’t dramatically increase the number of identified splice junctions, suggesting that 17M reads already reached or at least is close to the saturation point (Supplementary Figure S3B); (iii) the same patterns have been observed using all available reads (Supplementary Figures S3C,D). Thus, we believe that the main conclusions of the work are not affected by the relatively low sequencing depth or stochasticity from the random sampling. However, further studies using similar datasets with higher number of reads can provide stronger evidence for this. The species-specific AS clustering pattern was also found among vertebrate species that span ∼350 million years of evolution (Barbosa-Morais et al., 2012; Merkin et al., 2012), indicating that this might be universal among eukaryotes. Interestingly, in vertebrates, some tissues, such as brain, testis, heart and muscle still showed a strong tissue-specific splicing signature, despite the dominant species-specific splicing background (Barbosa-Morais et al., 2012; Merkin et al., 2012). Although all three tissues (root, leaves, and flowers) used in our study did not show such strong tissue-specific splicing signatures, some other plant tissues might. For example, the transcriptomes of sexual tissues are substantially different from those of vegetative tissues, and anthers harbor the most diverged specialized metabolomes (Yang and Wang, 2013; Li et al., 2016). Future studies that include transcriptome data of much more fine-scaled tissue samples will provide new insights on this aspect.

AS events that resulted in transcripts with PTC, are coupled with nonsense-mediated decay NMD. They are more conserved than the AS that do not generate PTC-containing transcripts in plants (Figure 3A and Supplementary Figure S10). Consistently, among six ultra-conserved AS events across different plant species including the spreading earth moss, five produced +PTC transcripts, indicating that AS+PTC might be more important than it was previously thought. Previous studies showed that all human serine/arginine-rich (SR) genes and some SR genes in plants produce AS resulted in +PTC transcripts (Kalyna et al., 2006; Lareau et al., 2007; Filichkin et al., 2010; Palusa and Reddy, 2010). Furthermore, the junction regions that contain AS+PTC in numerous splicing factors (SFs) are ultra-conserved between different kingdoms and the loss of the ancient AS+PTC in paralogs through gene duplications were repeatedly replaced by newly created distinct unproductive splicing (Lewis et al., 2003; Lareau et al., 2007; Lareau and Brenner, 2015). Similar to these previous works, our results are consistent with the hypothesis that the unproductive splicing coupled with NMD can be a functional process that influences the abundance of active proteins at a post-transcriptional level.

One caveat from our analysis on the conservation of AS+PTC events is that we focused on only a subset of AS events, due to methodological challenges of associating AS events with specific transcripts and annotating PTC. Identifying and annotating AS+PTC events from RNA-seq data is computationally challenging, to reduce the false positives, we applied stringent filtering parameters, and only focused on the transcripts that can be uniquely associated with a single AS event. Although the same filtering parameters were used for all of the AS events and the observed pattern is unlikely to be the result of such filtering, it remains unclear whether this pattern represents all AS events. Future studies that combine full-length transcript (Wang B. et al., 2016) and long reads sequencing technologies will reduce the computational complexity and errors involved in associating AS events with transcripts and may provide more robust analysis on the evolution and conservation of AS+PTC in plants.

Among the five investigated plant species, the distance between the 5′/3′ nearest alternative SS and the authentic SS is the main determinant that distinguishes AltD/AltA from constitutive splicing (Figure 4 and Supplementary Data Sheet S1). For a given spliced junction, the likelihood of AS decreases with an increased distance between the authentic and nearest alternative SS (Figure 4A,B). Interestingly, similar patterns were also found in mammals, in which, the closer the alternative SS was to the authentic SS, the more likely it was used for AS (Dou et al., 2006; Rosenberg et al., 2015). The frequency of AltA also decreases with the increased distance between the authentic SS and nearest inter-AG dinucleotide. This result is consistent with the pattern found in humans in that only closely located AGs ( < 6 nt) can effectively compete with the authentic SS and the distance between branch site and the first downstream AG can affect the 3′ SS selection (Chiara et al., 1997; Chua and Reed, 2001). Although, the BS in plants is not well studied and BS was not identified in ∼30% of junctions, a similar effect of inter-AG distance on AltA in both plants and mammals indicates that the mechanisms of generating AS, at least for AltA, might be similar between these two kingdoms.

While the deep learning model for AltA achieved high precision and specificity among five species (AUC > 0.9, indicating high precision and high specificity), the models for AltD performed less well, although still performing better than by chance (AUC > 0.75, Figure 4C,D and Supplementary Figure S16). This indicates that additional determinants that contribute to the regulations of AltD were not detected by our method. It is known that the mechanisms involved in AltD are more complex than in AltA. For example, in both human and mouse, while both the presence and quantity of exon splicing enhancer and exon splicing silencer are important for generating AltD (Koren et al., 2007). While binding sites of splicing factors can also be important, AltA is mainly affected by the competition of closely located AG dinucleotide by a scanning mechanism for the downstream sequence of the branch site polypyrimidine tract (Smith et al., 1989; Smith et al., 1993; Chiara et al., 1997; Chua and Reed, 2001). Furthermore, it is known that NAGNAG (N is any nucleotide), which is a subset of SS for AltA that are separated by three nucleotides, are enriched in genes encoding DNA-binding proteins in both plants and animals (Vogan et al., 1996; Iida et al., 2008; Schindler et al., 2008). These results suggest that splicing regulatory elements (SREs) may play more important roles in the proper selection of alternative SS in AltD than AltA. This may also explain why the junction size contributed more in the AltD model than in the AltA model (Supplementary Data Sheet S1), since larger junction size might increase the likelihood of introducing intronic SREs. Although a few candidate sequence motifs were identified using the enrichment analysis, none of them significantly contributed to the model predictions. Two non-exclusive possibilities may partially explain this failure. First, the identified motifs are not involved in affecting splicing processes, although their density was significantly different between constitutively and alternatively spliced junctions. Second, they might be essential for tissue-specific AS, which likely did not contribute to the overall AltD prediction based on all three tissues. Future studies using different approaches to investigate the alteration of AS by introducing millions of random hexamers into specific regions of a gene junction in a plant then measuring the consequences of splicing, may allow us to more reliably detect splicing regulators of AltD in plants.

Although we found that both junction size and SS for IR junctions were different between the constitutively and alternatively spliced junctions (Supplementary Figure S13), the identified features did not improve the AS prediction from that occurring by chance. There are three non-exclusive possible reasons. First, the expression level of IR is usually low and therefore requires high sequencing depth for their detection (Supplementary Figure S19). It is possible that the sequencing depth of the transcriptome data used in this study was not sufficient to detect all of the IR junctions. In such case, many true IR junctions may not have been considered as IRs in our analysis, which reduced prediction precision and power. Second, a recent study showed that a subset of IR junctions – exitron – has different features from regular IR junctions (Marquez et al., 2015). Thus their determinant might also be different. Third, previous work showed that a large proportion of IRs (76.5%) identified from RNA-seq result from incompletely spliced pre-mature mRNA (Zhang et al., 2015), thus increasing the false positives of IR. Future studies that sequence transcriptomes of different tissues among species using polyribosomal RNA-seq technology (Zhang et al., 2015) in high depth will likely reveal the mechanisms underlying IR regulations in plants.

For both AltA and AltD, their divergence between closely related species was likely due to variations in the key sequence determinants near the SS (Figure 5 and Supplementary Figures S17, S18) and the key sequence determinants such as distance to authentic SS and *cis*-elements (branch site, polypyrimidine tract, UA-rich tract for AltA), which are all located within intronic regions. Intron sequences diverge faster than protein coding regions (Mattick, 1994; Hare and Palumbi, 2003), therefore, the process that likely have contributed to the species-specific gains and losses of AS among different lineages to produce species-specific AS profiles in plants. For example, a decreased distance between alternative SS and authentic SS as a result of a short deletion of the intron sequence could lead to a gain of AS at the junction, and as the consequence it is likely to be shared among different tissues. Consistently, in vertebrates, the mutations that affect intronic SREs were shown to be the main factor that resulted in the dominant species-specific splicing pattern (Merkin et al., 2012). However, it is unclear whether the observed changes of AS were neutral or under selection, because defining the null model of the AS evolution remains challenging. Furthermore, our data cannot exclude the possibility that the species-specific *trans*-factors, such as the SR protein families, which have distinct numbers of homologs among species (Supplementary Figure S20; Iida and Go, 2006; Isshiki et al., 2006; Ling et al., 2015), may have also contributed to the divergence of AS among different species (Ast, 2004; Barbosa-Morais et al., 2012). For example, there are 38 SR homologs in soybean, which is much higher than the number of SR homologs in other plant species (Supplementary Figure S20). Such species-specific expansion of certain SR families may contribute to the relative unique AS pattern of soybean (Figure 1A,B).

We also investigated other factors that were hypothesized to affect AS evolution, such as gene duplication, DNA methylation and TE insertion (Sorek et al., 2002; Su et al., 2006; Flores et al., 2012). However, with the exception of TE insertions, the effects of which were found to be species-specific, most of the tested factors did not show significant effects on the levels of AS conservation between closely related species (Supplementary Figure S18 and Supplementary Data Sheet S2). The species-specific effects of TE on the AS conservation were likely due to the different abundance of TE insertions in the genomes of different species (Hu et al., 2011; Tomato Genome Consortium, 2012; Slotte et al., 2013; Sierro et al., 2014), suggesting genomic composition of each species might also affect the evolutionary alteration of AS.

## Conclusion

We found that the divergence of AS profile among species is associated with massive gains and losses of AS in each lineage, while a group of AS that generate PTC-containing transcripts were highly conserved even among very distantly related plants. The alteration of a few key sequence determinants of AltA and AltD, all located in the intron region, likely contributed to the divergence of AS among closely related plant species. These results provide mechanistic insights into the evolution of AS in plants and highlight the role of post-transcriptional regulation of a plant’s responses to environmental stresses.

## Author Contributions

ZL and SX designed the research. ZL, TB, and SX performed the experiments and analyzed the data. ZL, IB, and SX wrote the manucript.

## Conflict of Interest Statement

The authors declare that the research was conducted in the absence of any commercial or financial relationships that could be construed as a potential conflict of interest.

## Footnotes

1 http://www.ncbi.nlm.nih.gov/sra
