# Supplementary material for: Evolution of Alternative Splicing in Eudicots
Source: Front Plant Sci. 2019 Jun 12;10:707. doi: 10.3389/fpls.2019.00707 (PMC6581728; doi:10.3389/fpls.2019.00707)
Supplement: Supplementary file 2 [file Table_2.docx]

**Table S2. RNA-seq coverage and alignment statistics of Brassicaceae**
