# Supplementary material for: Evolution of Alternative Splicing in Eudicots
Source: Front Plant Sci. 2019 Jun 12;10:707. doi: 10.3389/fpls.2019.00707 (PMC6581728; doi:10.3389/fpls.2019.00707)
Supplement: Supplementary file 4 [file Image_1.pdf]

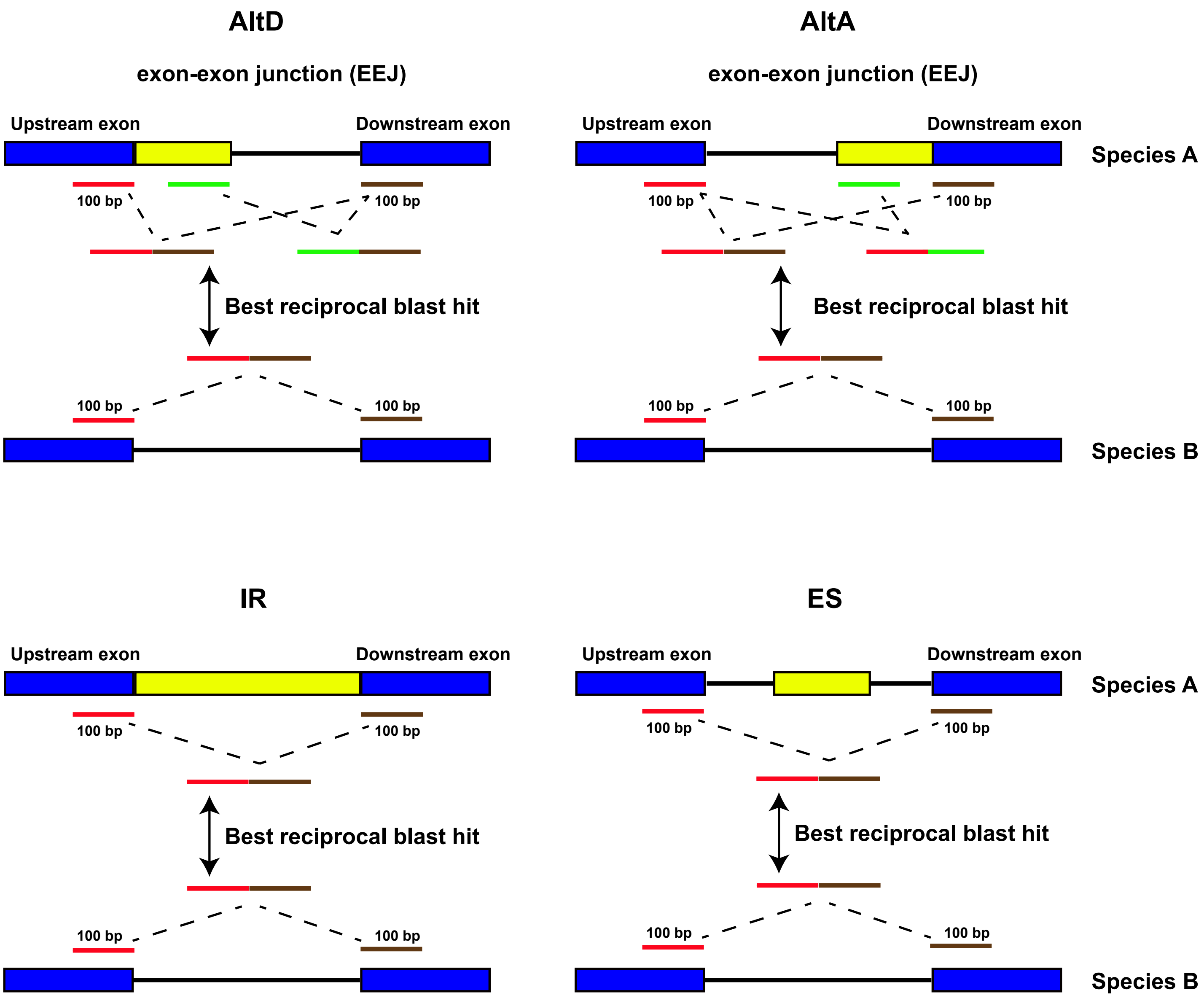

Figure S1. The method of identifying conserved exon-exon junctions (EEJs). The compared EEJs must belong to one-to-one orthologous between two species, and 100 bp sequence from the flanking upstream and downstream exons of each junction were combined (in total 200 bp sequence) to represent the EEJ. In addition to the outer EEJ, if inner EEJs exist due to AS, all EEJs sequences were used for comparison, and only EEJ pair that are the best reciprocal blast hit between two species were regarded as conserved EEJs. All scenarios in the figure assume the AS exists in species A but not in species B. (AltD: alternative 5' donor site; AltA: 3' acceptor site; IR: intron retention; ES: exon skipping).

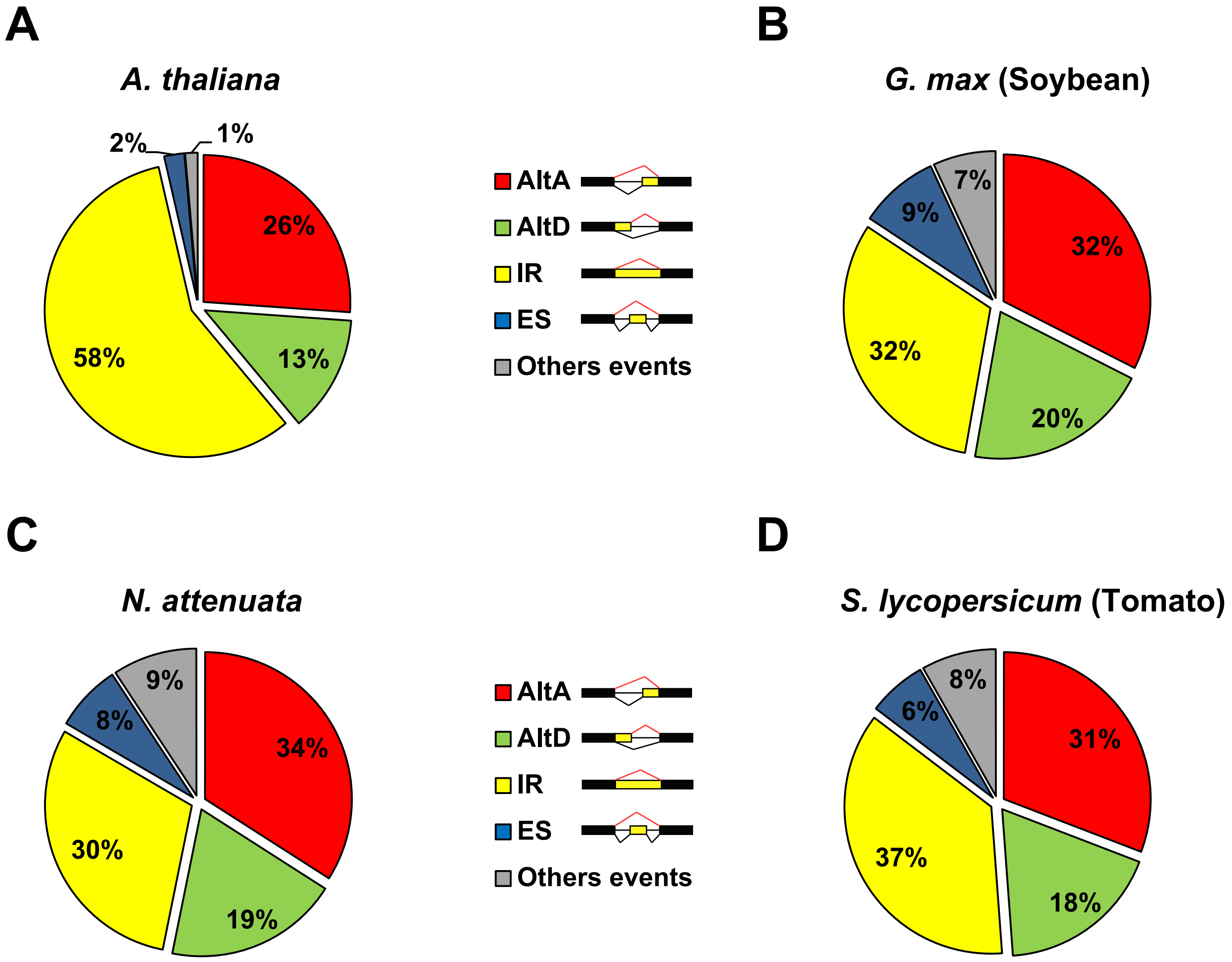

Figure S2. The distribution of different alternative splicing (AS) types in *A. thaliana* (A) , *G. max* (B), *S. lycopersicum* (C) and *N. attenuata* (D). IR: intron retention; AltA: alternative 3' acceptor site; AltD: alternative 5' donor site; ES: exon skipping.

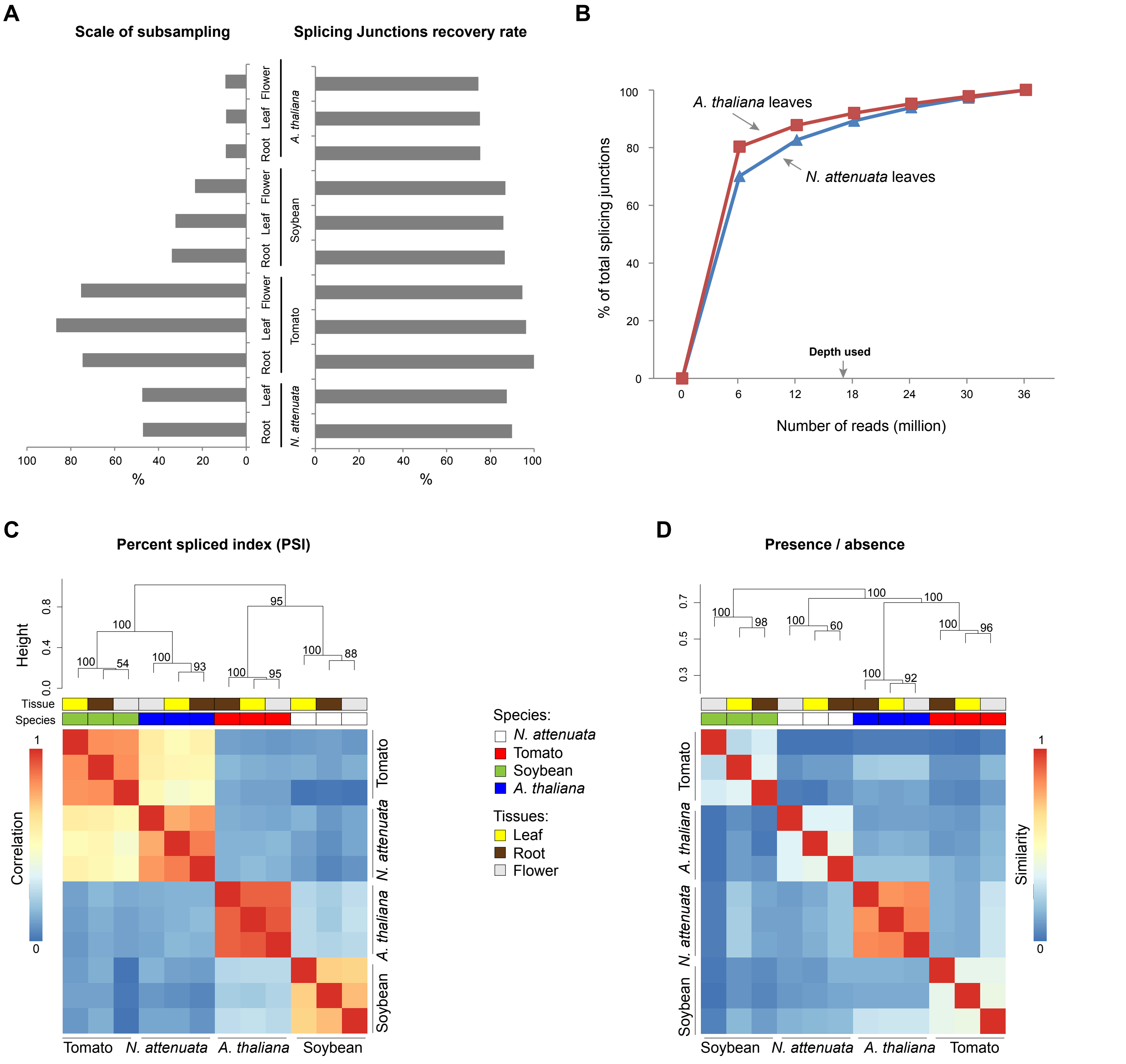

Figure S3. The subsampled 17M reads are sufficient to reveal the AS evolution in the studied species. (A), the scale of subsampling and the splicing junction recovery rate are shown in left and right, respectively. The scale of subsampling is calculated as the ratio of 17M reads relative to the total number of available reads, and the splicing junctions recovery rate is calculated as the number of splicing junctions that are identified from 17M reads relative to the number identified from total reads. (B), the saturation curve of detecting splicing junctions with different sequencing depths. X-axis refers to the number of sub-sampled reads. Y-axis refers to the percentage of detected splicing junctions relative to the total number of splicing junctions detected from 36 million reads. Leaf samples from *N. attenuata* and *A. thaliana* are shown in blue and red colors, respectively. (C) and (D), heatmaps depict species-specific clustering based on the full dataset (all available reads) using PSI values (C) and presence and absence of AS (D). In total, junctions from 1,306 (C) and 5,002 (D) orthologous genes were used for the clustering. Numbers present in each branch node represent the approximately unbiased bootstrap value calculated from 1,000 bootstrap replications. The color code above each heatmap represents species, tissue, and treatments.

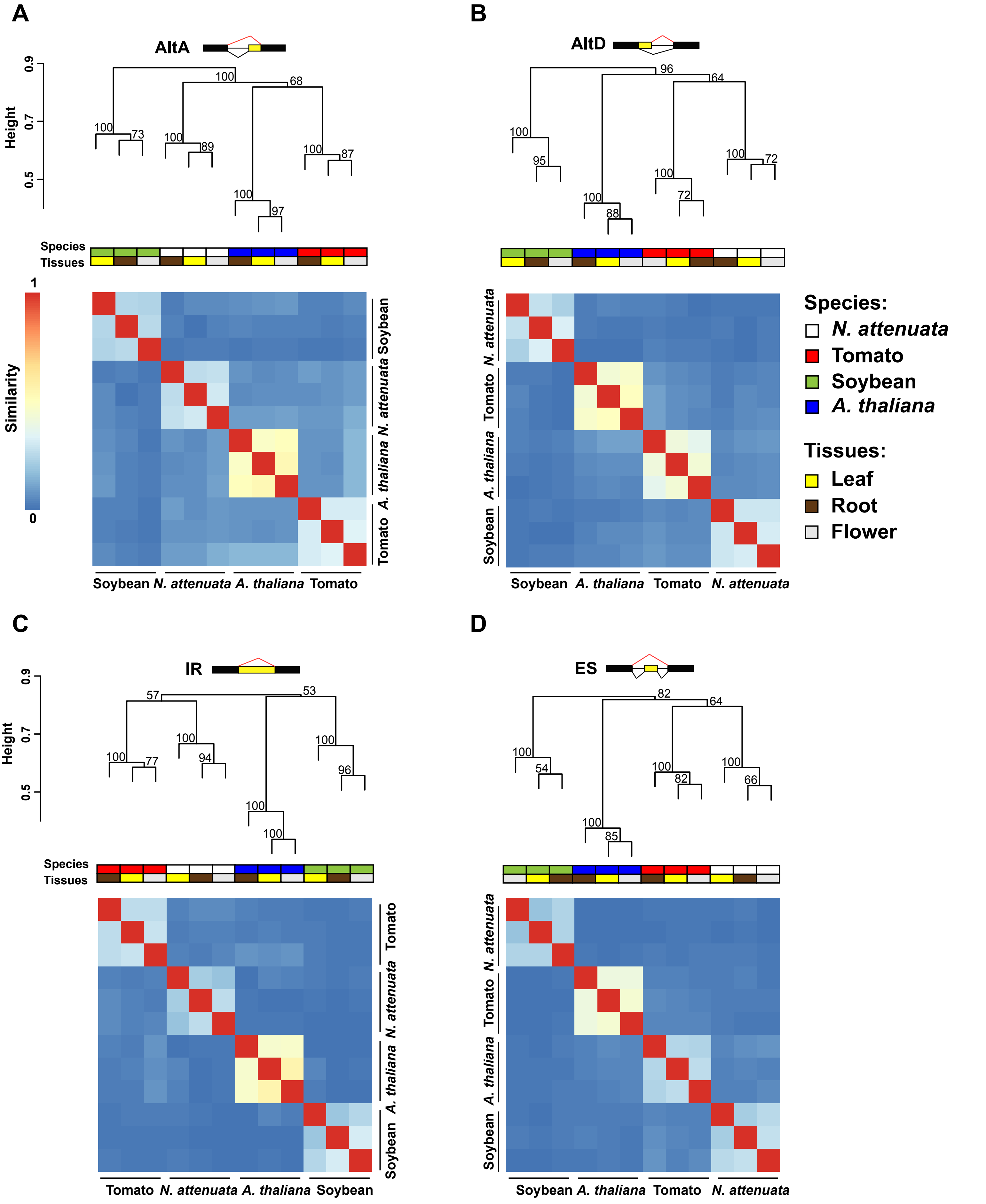

Figure S4. Different types of AS all show species-specific clustering pattern. Heatmaps depict species-specific clustering based on presence and absence of AS from one-to-one orthologous genes present among all four species (n = 3,857). (A), alternative 3' acceptor site (AltA). (B), alternative 5' donor site (AltD). (C), intron retention (IR) and (D) exon skipping (ES). Numbers at each branch node represent the approximately unbiased bootstrap value calculated from 1,000 bootstrap replications. The color code of the heatmap represents species, tissue, and treatments.

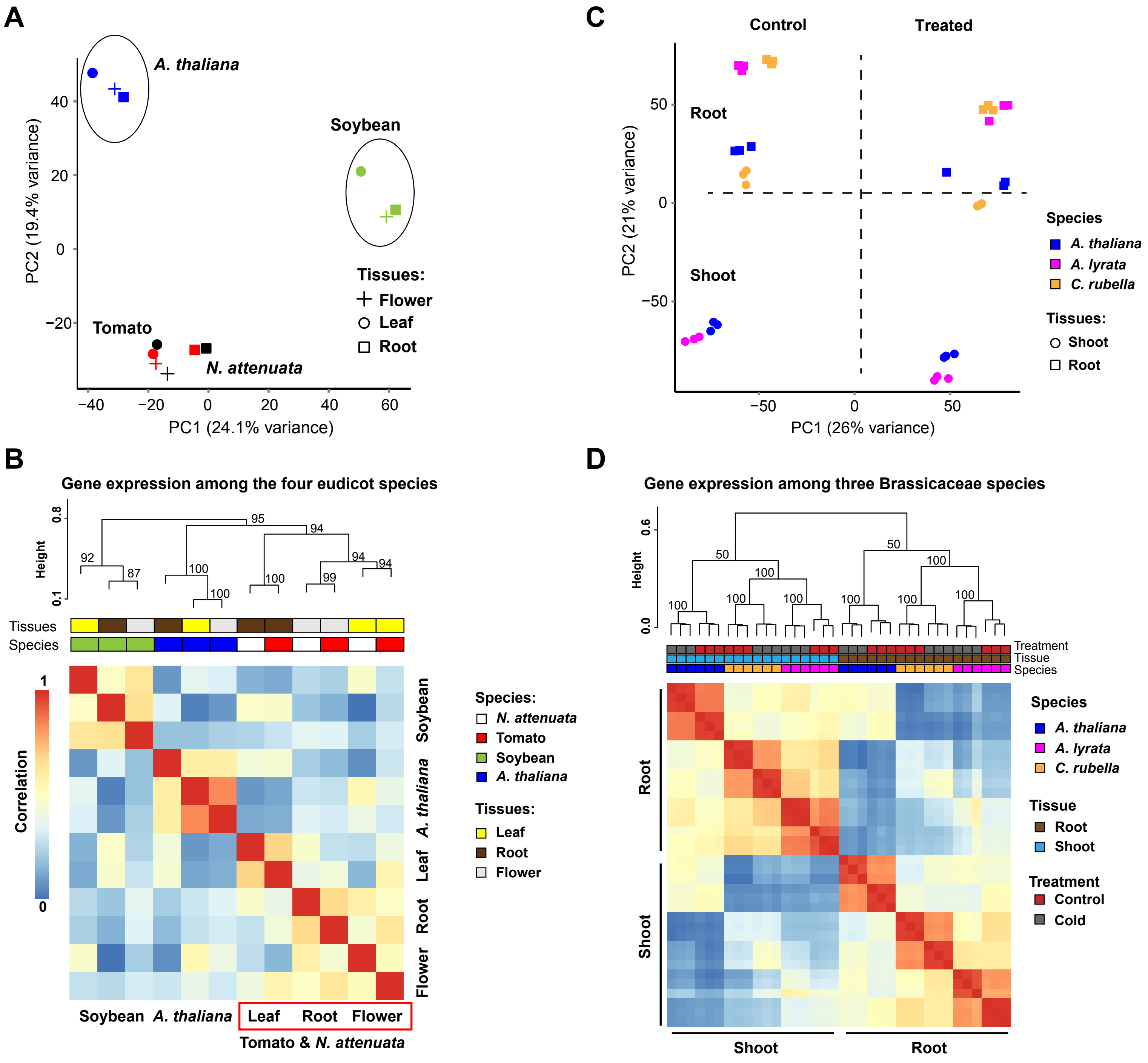

Figure S5. Similarity of gene expression (GE) among different plant species. (A) and (B), PCA and complete linkage heuristic clustering of different tissues based on normalized gene expression of one-to-one orthologues genes among the four eudicot species (n = 5,745). (C) and (D), PCA and complete linkage heuristic clustering of different organs based on normalized gene expression of one-to-one orthologues among the three Brassicaceae species (n = 15,969). Numbers at the branches represent the approximately unbiased bootstrap value calculated from 1,000 bootstrap replications.

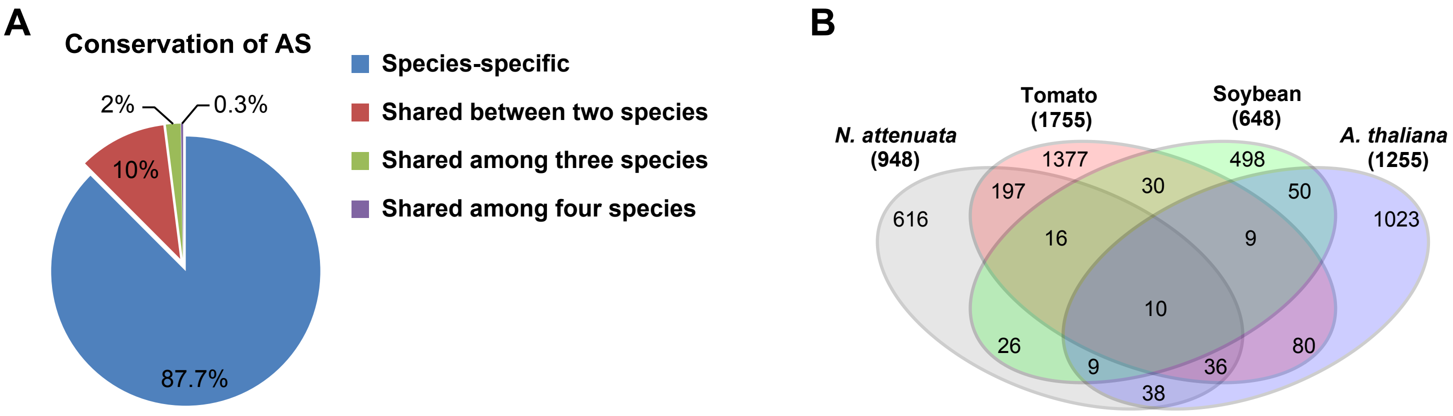

Figure S6. Conservations of alternative splicing among the four eudicot species. (A) Percentage of AS that are conserved among species. The majority of AS events are species specific. (B) A venn-diagram depicts the conservation of 4,015 AS events from the exon-exon junctions that are at least conserved between two species.

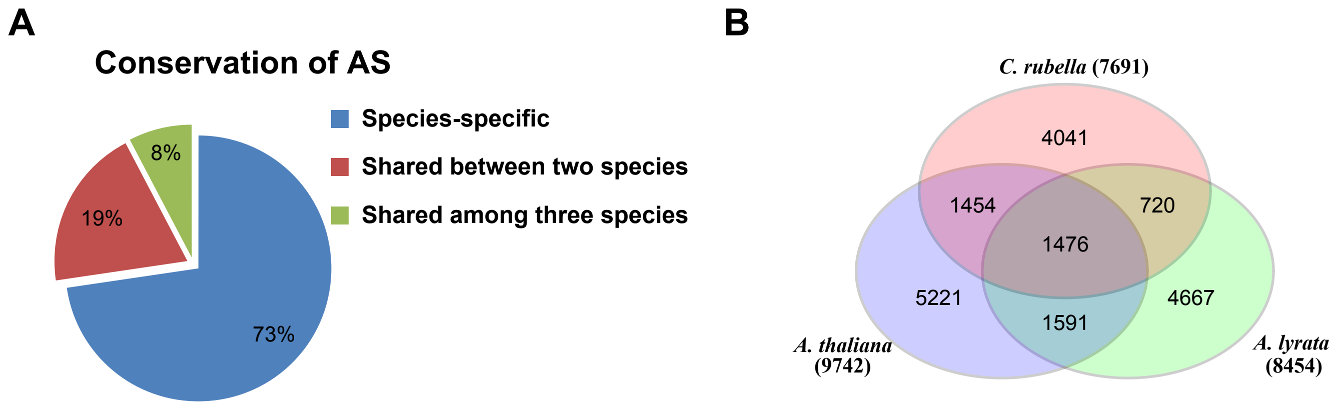

Figure S7. Conservation of alternative splicing (AS) among the three Brassicaceae species. (A) The percentage of AS that are conserved among species. The majority of AS events identified are species specific. (B) A venn diagram depicts the distribution of 19,170 AS events at the exon-exon junctions that are conserved or species specific.

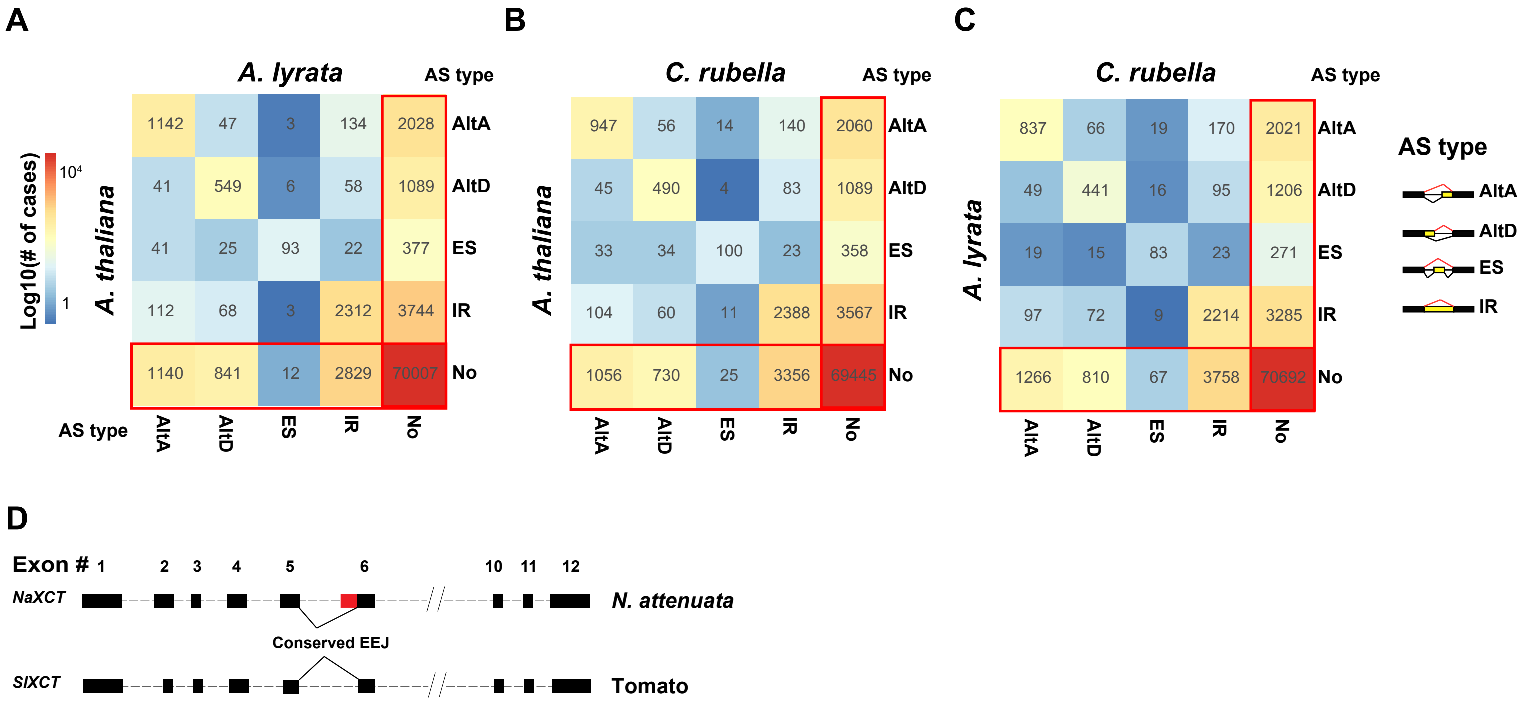

Figure S8. The transition spectrum among different types of alternative splicing (AS) between additional species pairs and two examples. (A) *A. thaliana* vs *A. lyrata*, (B) *A. thaliana* vs *C. rubella*, (C) *A. lyrata* vs *C. rubella*. Only the exon-exon junctions (EEJs) with at most one AS event were considered. The color of each grid indicates the abundance of AS events. AltA: alternative 3' acceptor site; AltD: alternative 5' donor site; ES: exon skipping; IR: intron retention. (D) shows an example of AS transition. While an AltA event was found in *XCT* in *N. attenuata*, which was also confirmed by RT-PCR in our previous work, no AS was found at its orthololgous junction in tomato (*Solyc01g111140.2.1*).

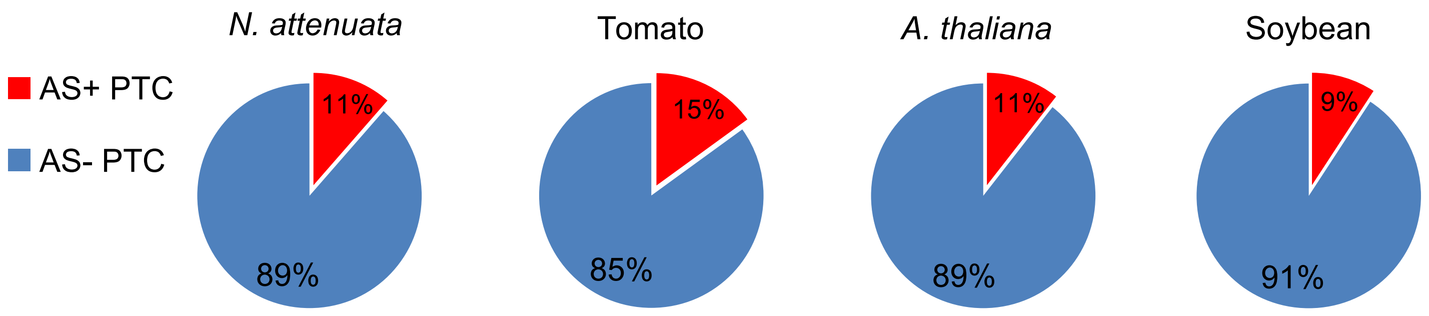

Figure S9. The proportion of alternative splicing (AS) that generates premature termination codons (PTC) containing (+PTC) or non-PTC containing (-PTC) transcripts in *N. attenuata*, tomato, *A. thaliana* and soybean.

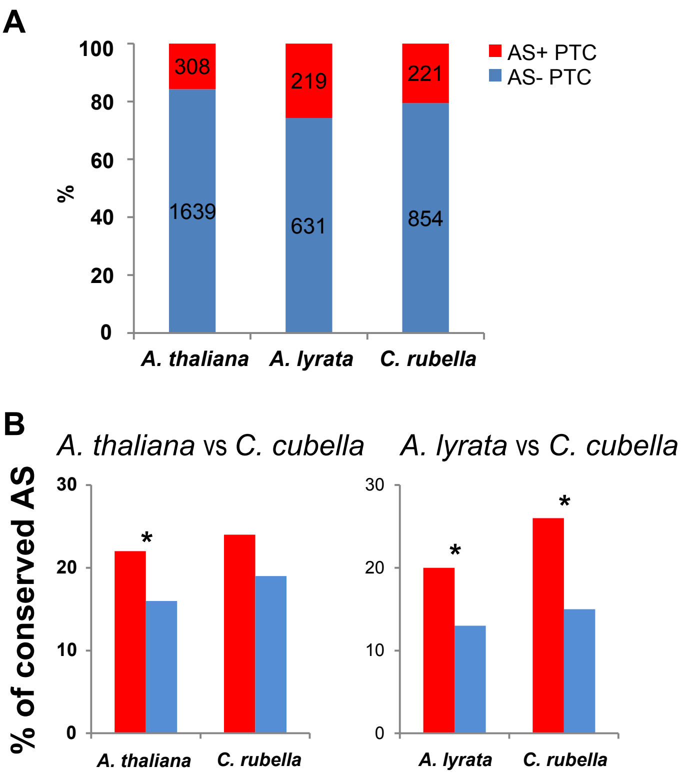

Figure S10. Comparison between the AS events that generate premature termination codons (PTC) containing transcripts (+PTC) and the AS events that do not generate PTC containing transcripts (-PTC) between species pairs in Brassicaceae. (A) The number and relative portions of AS+/- PTC in *A. thaliana*, *A. lyrata* and *C. rubella*. (B) The percentage of AS+ and AS- PTC that are conserved between species pairs. Asterisks indicate the significance as determined by Fisher's exact test ($P < 0.05$). Between *A. thaliana* and *C. rubella*, the conservation difference between AS+ PTC and AS- PTC AS in *C. rubella* is marginal ($P = 0.09$).

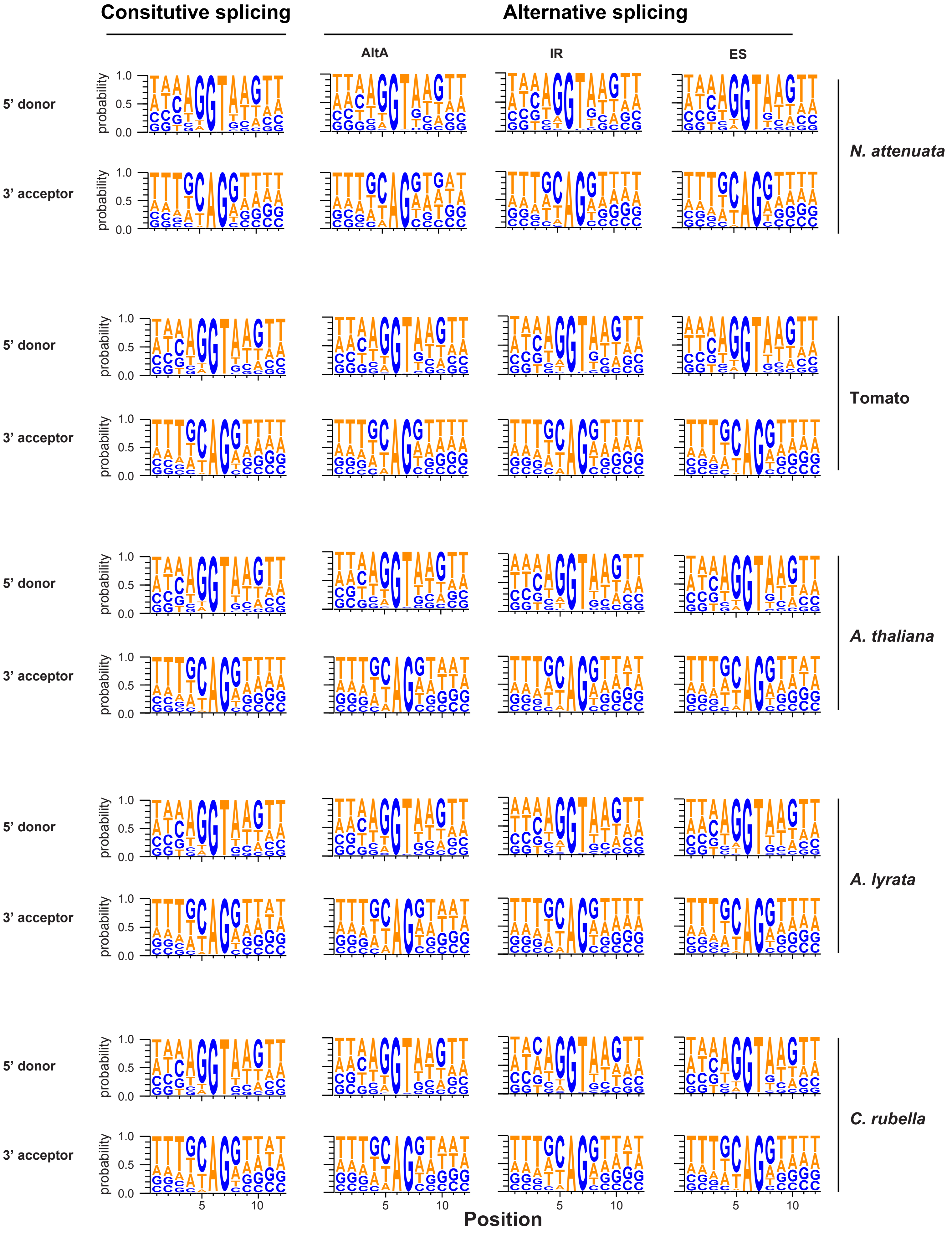

Figure S11. The nucleotide composition of constitutive and alternative splice sites and their surrounding sequences among five plant species.

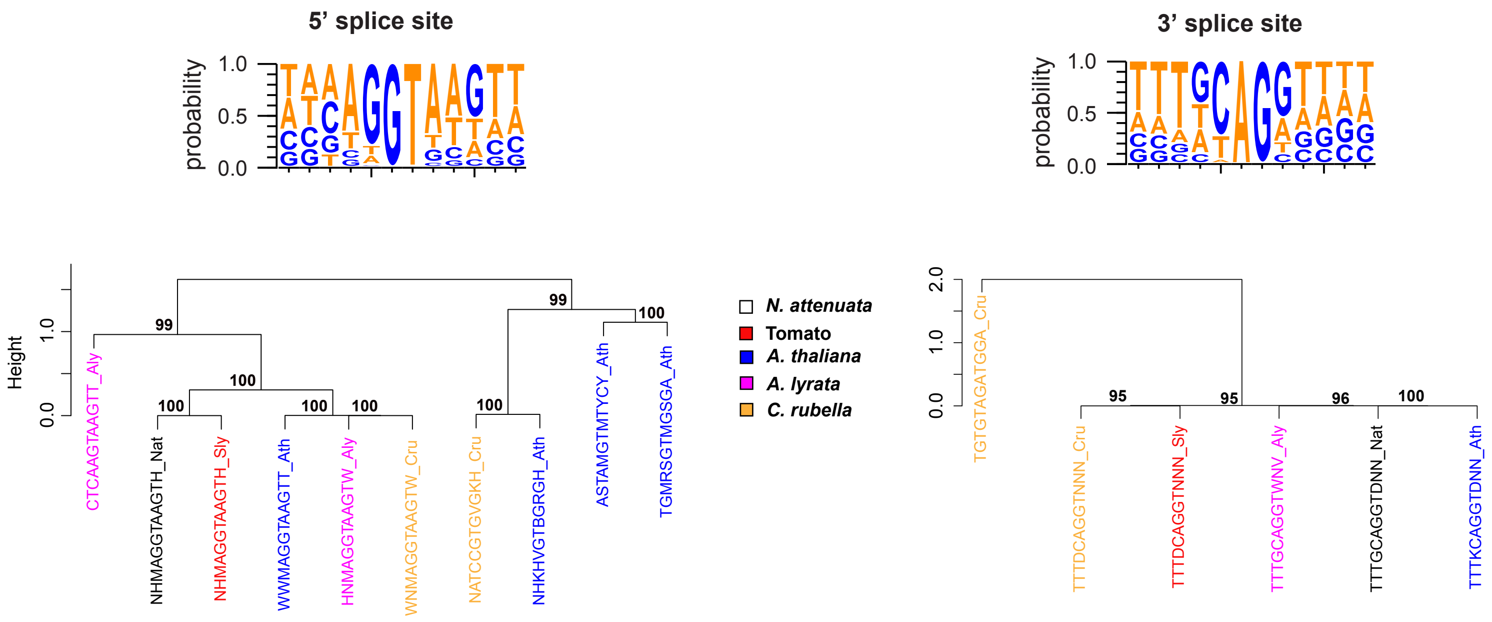

Figure S12. The complete linkage hierarchical clustering of splice site motifs among different plant species. The distance was measured by the Pearson correlation. The number at each branch node represents the approximately unbiased bootstrap value calculated from 1,000 bootstrap replications.

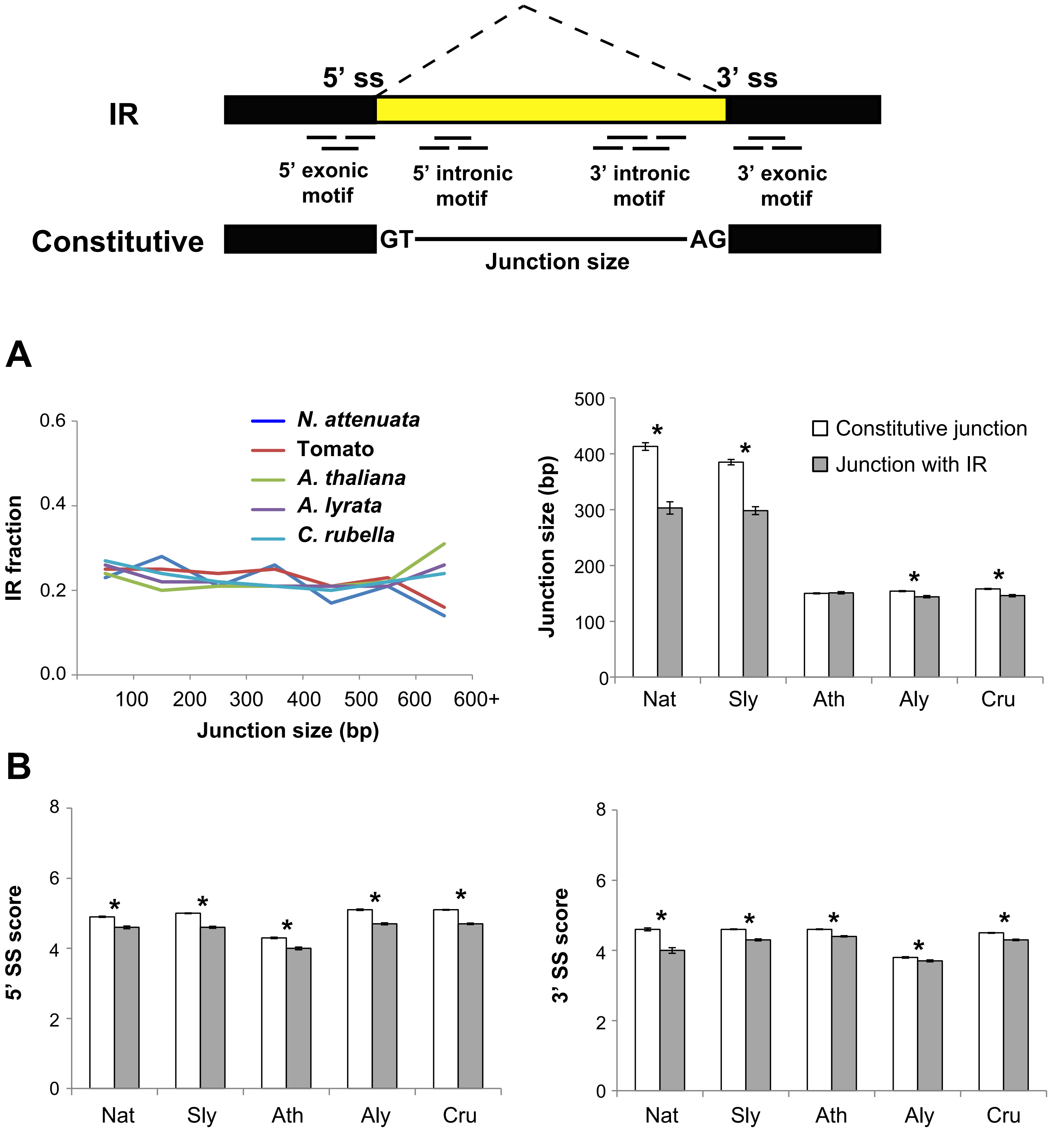

Figure S13. The determinants of intron retention (IR) in plants. (A), the frequencies of IR for junctions with different size and the average size between the constitutively spliced junction and the junction with IR. (B), the average 5' and 3' splice site score between the constitutive junctions and the junctions with IR. The asterisk indicates a significant difference determined by Student's t-test ($P < 0.05$) and the error bars depict standard error (SE).

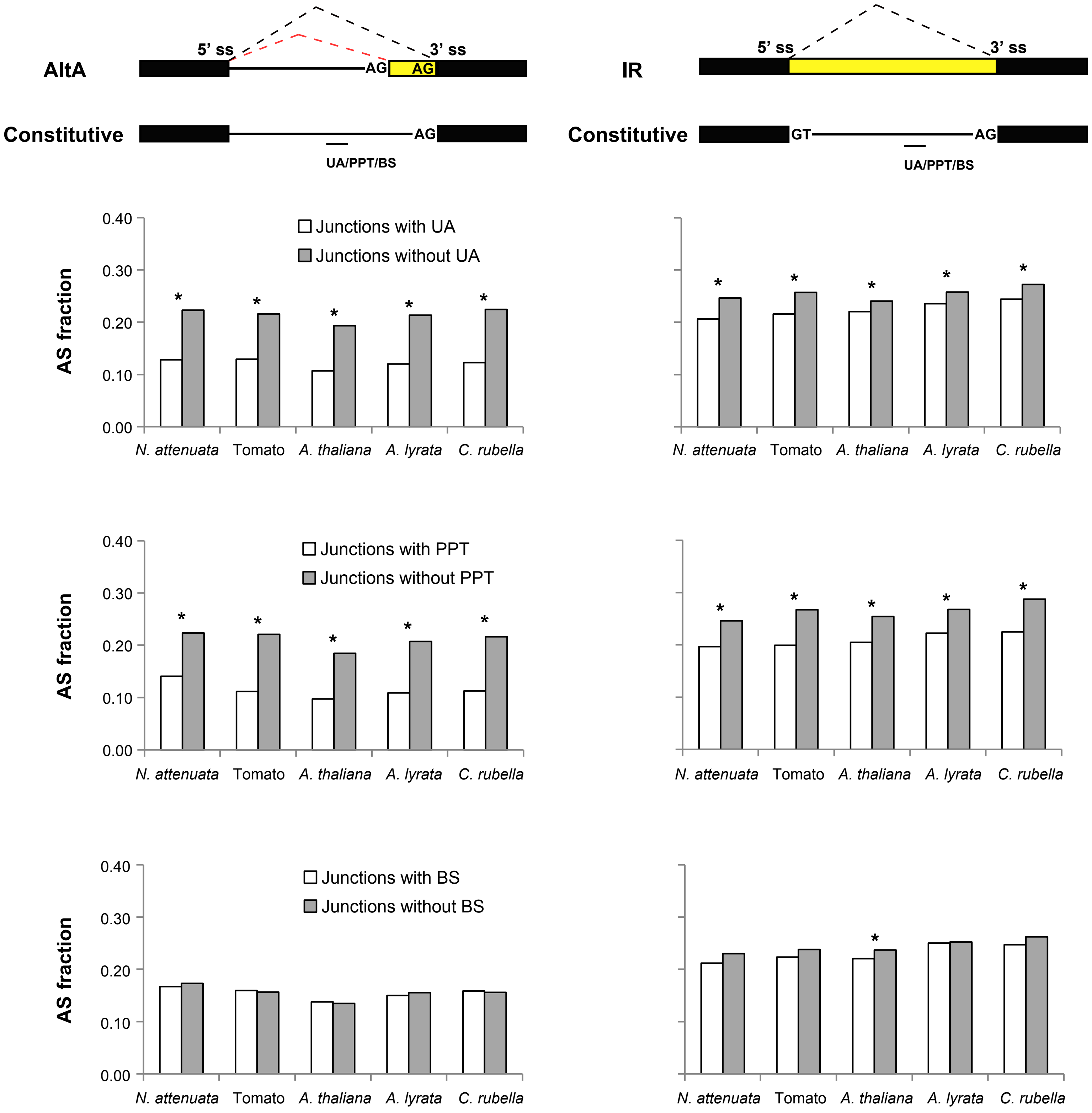

Figure S14. The effect of UA-rich, polypyrimidine tract (PPT) and branch site (BS) on alternative acceptor (AltA) and intron retention (IR) in plants. The frequencies of AltA and IR between junctions with and without each feature are shown. The asterisks indicate the significance determined by Fisher's exact test ($P < 0.05$).

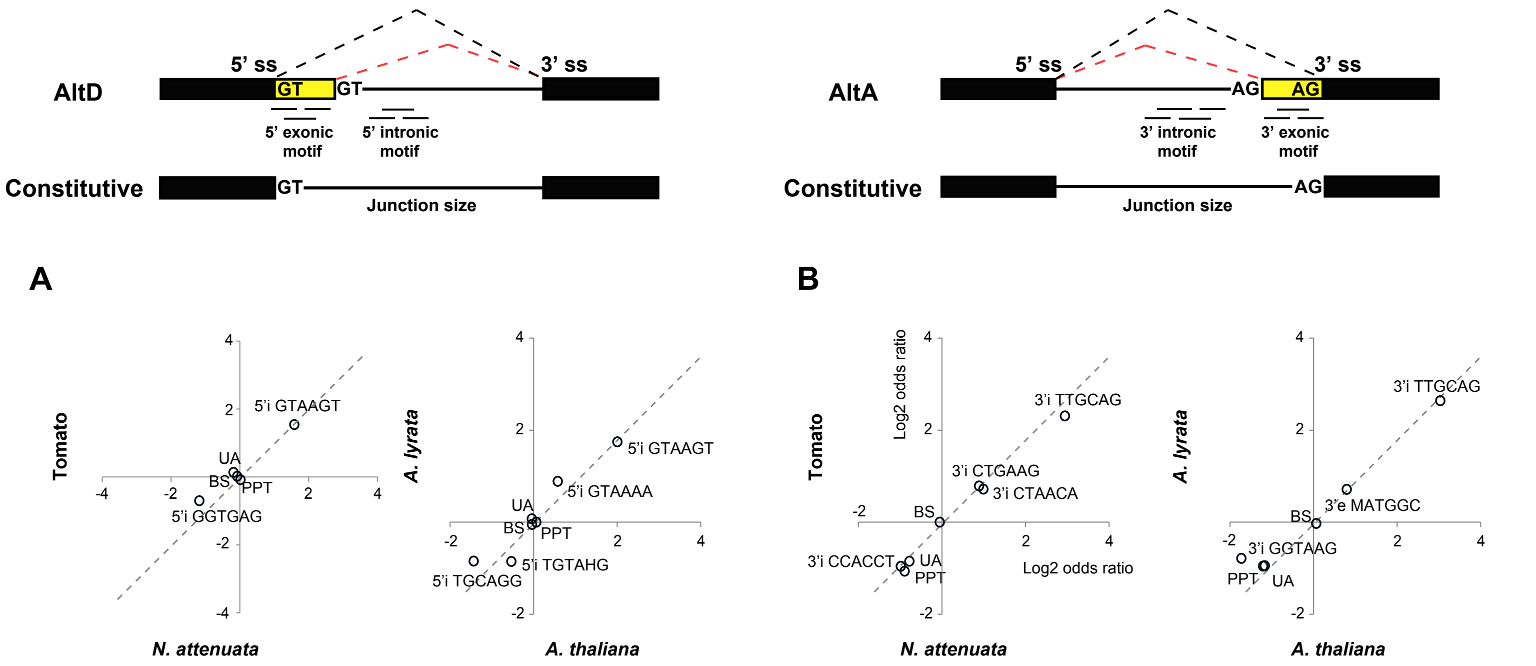

Figure S15. The enrichment of putative AS determinant features are largely conserved between closely related species. The enrichment of conserved 6-mer motifs, branch site (BS), UA and polypyrimidine tract (PPT) for AltD (A) and AltA (B) are compared between *N. attenuata* and tomato (left), and between *A. thaliana* and *A. lyrata* (right). X-axis and Y-axis are the level of enrichment in each species calculated as the ratio of the number of the feature found in the respective AS divided by the feature found in all junctions. The 6-mer motifs are shown as 5' or 3', intron (i) or exon (e) side, and motif sequences.

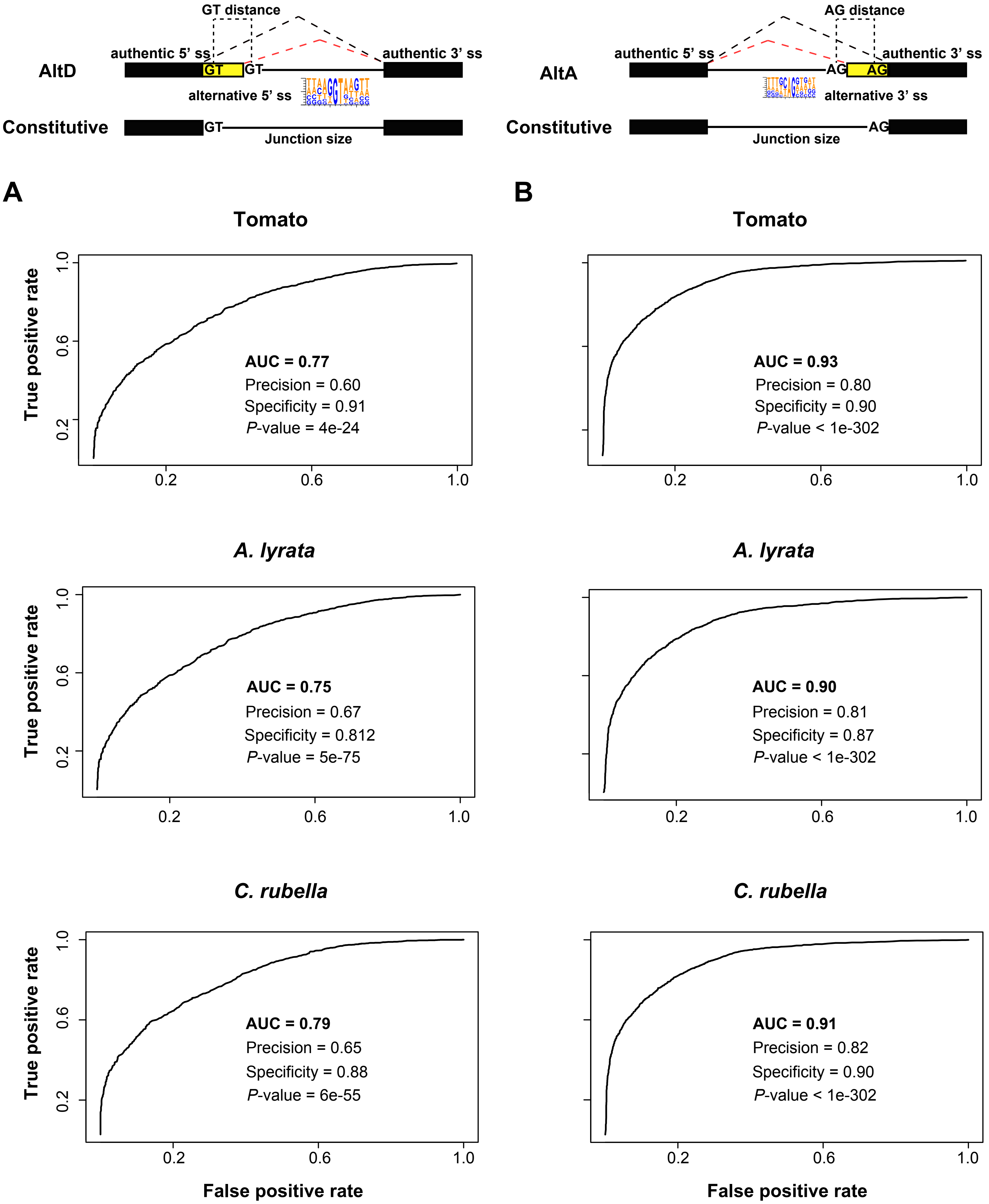

Figure S16. The area under the curve (AUC) from the deep learning models in tomato, *A. lyrata* and *C. rubella*. (A), alternative 5' donor (AltD). (B), alternative 3' acceptor (AltA). For each model, the model performance including AUC, accuracy, specificity and significance are also shown.

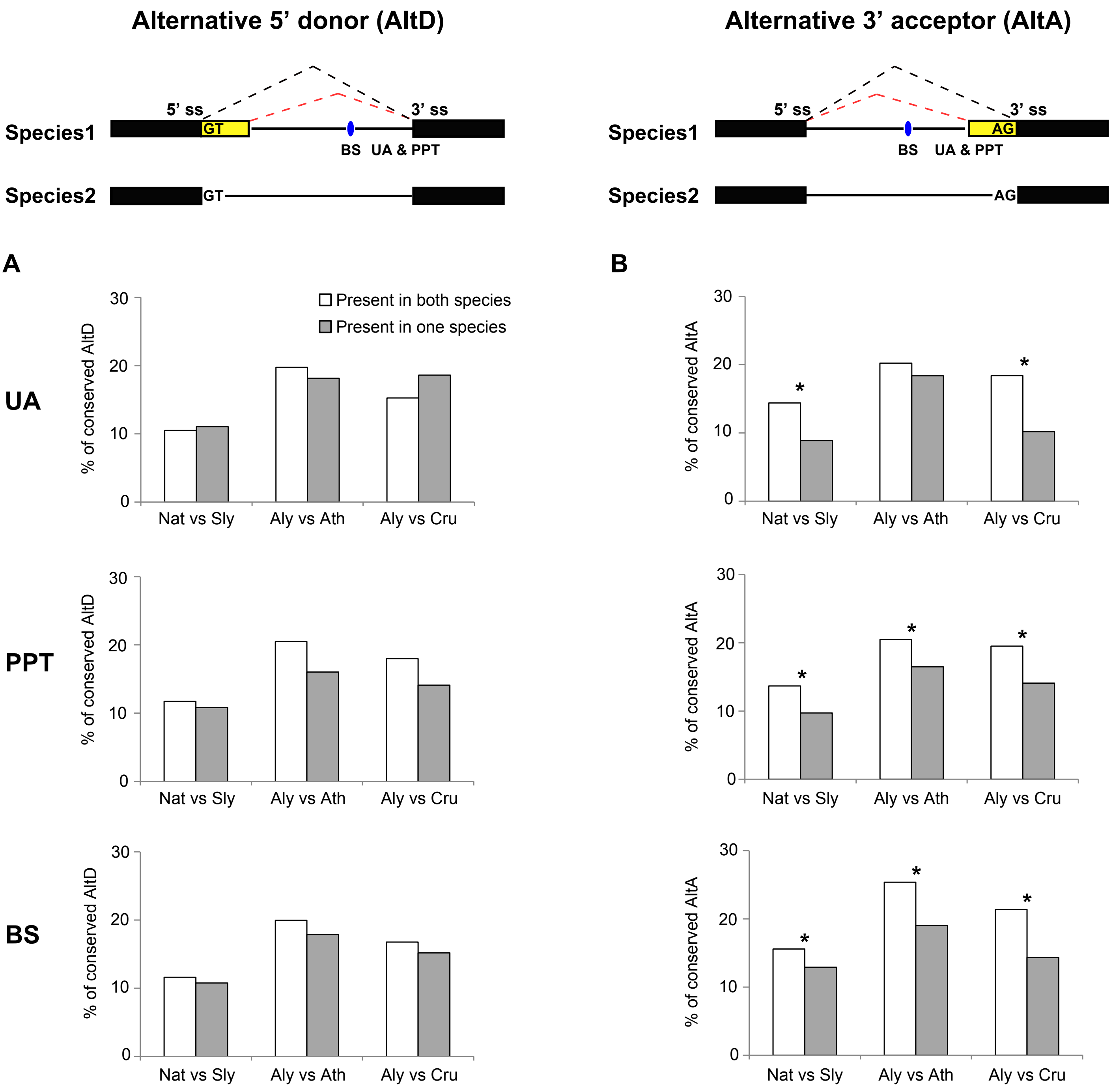

Figure S17. Changes of the cis-regulatory elements affect the divergence rate of AltD (A) and AltA (B) between species. The bar plots display the percentage of conserved AS in the group that UA, polypyrimidine tract (PPT) and branch site (BS) are different or same between two closely related species (Nat: *N. attenuata*, Sly: Tomato, Ath: *A. thaliana*, Aly: *A. lyrata*), the asterisks indicate the significance as determined by Fisher's exact test ($P < 0.05$).

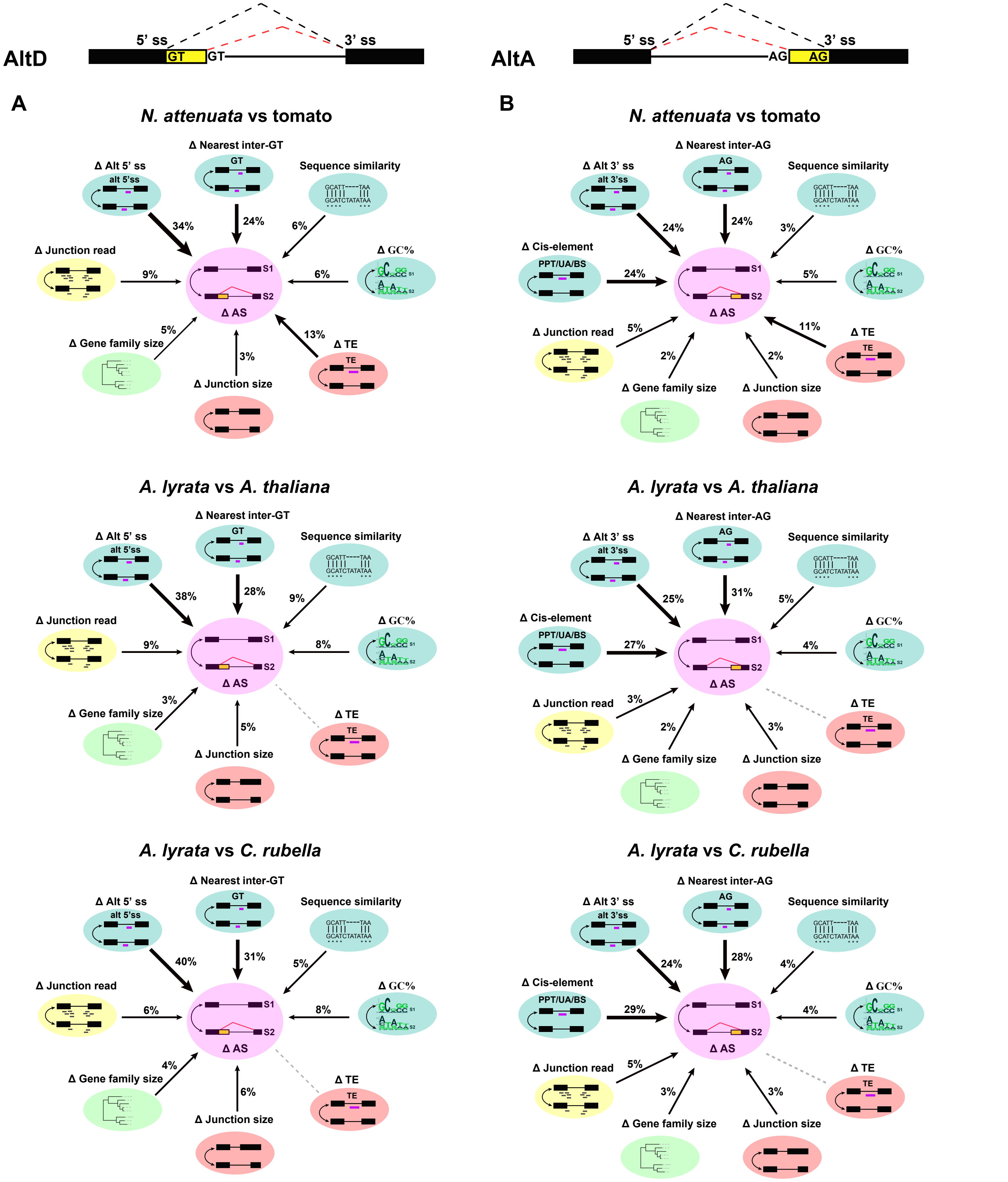

Figure S18. Factors that affect the rapid divergence of AS between plant species. Factors involved in AS divergence between *N. attenuata* and tomato, *A. lyrata* and *A. thaliana* and *A. lyrata* and *C. rubella* of (A) alternative 5' donor site (AltD) and (B) alternative 3' acceptor site (AltA) are shown. The number upon each arrow indicates the proportion of each factor that contributed to the model, the thickness of arrows are used to scale the contribution. Factors with no contribution are indicated by gray dotted lines.

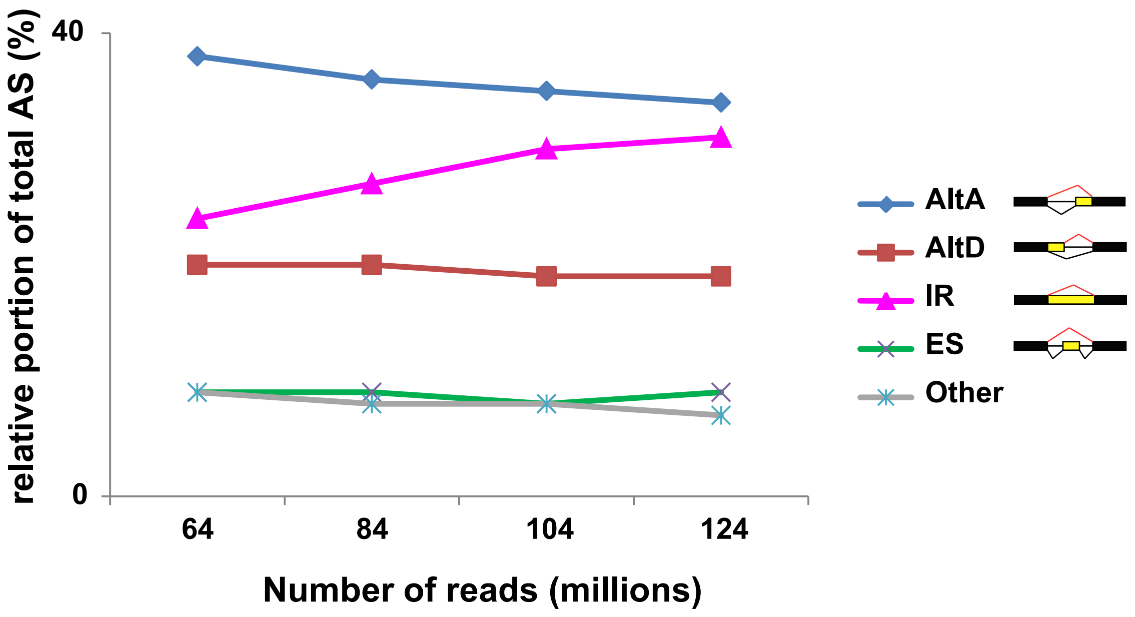

Figure S19. Sequencing depth affects the proportion of intron retention in leaf and root samples of *N. attenuata*. AltA: 3' acceptor site; AltD: alternative 5' donor site; IR: intron retention; ES: exon skipping.

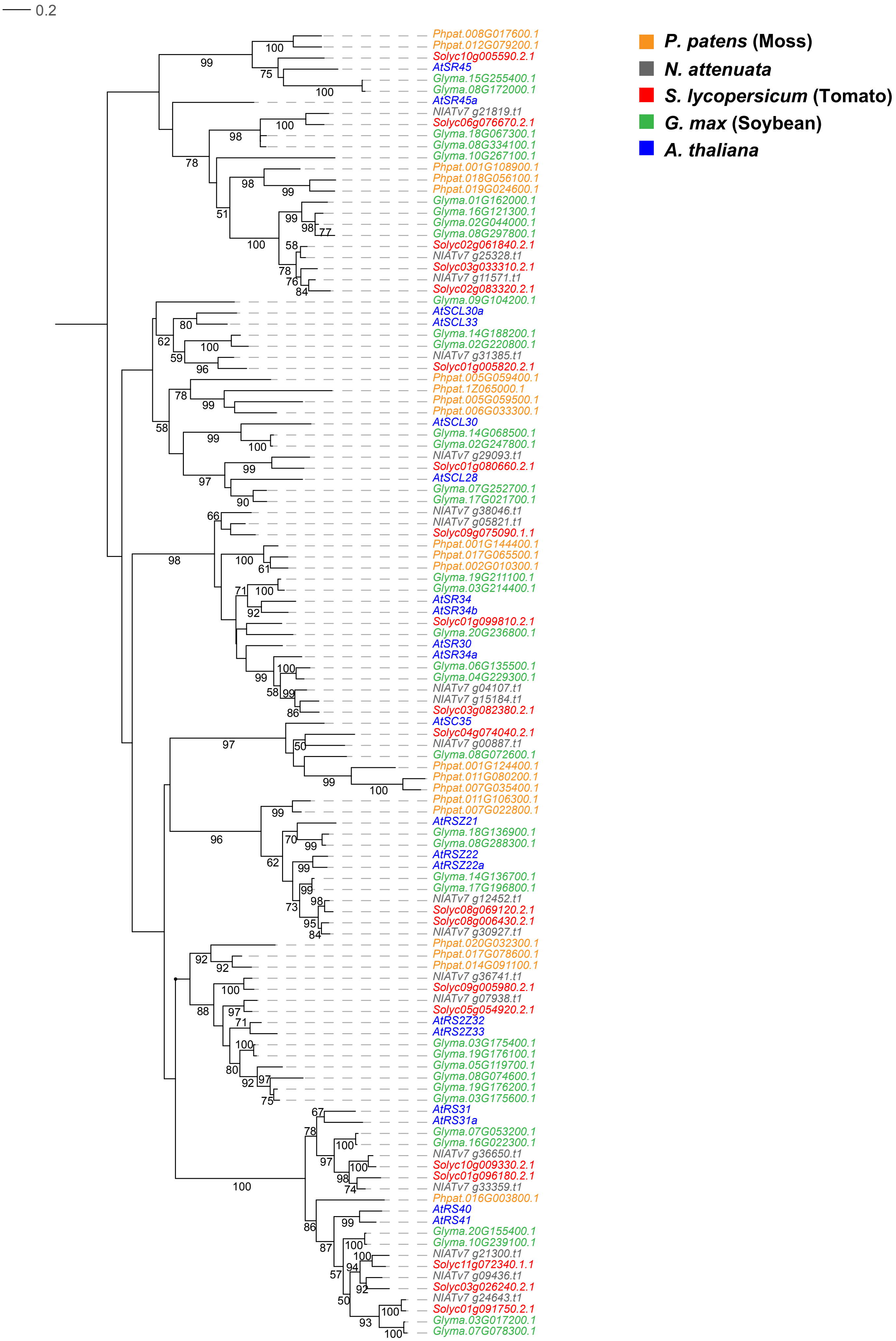

Figure S20. Phylogenetic tree of SR and SR-like genes in moss and the four eudicots species. The maximum likelihood method was used to construct the tree. Colors represent different species and only bootstrap values greater than 50 are shown at the branch.
